# Molecular signaling mechanisms behind polyphenol-induced bone anabolism

**DOI:** 10.1007/s11101-017-9529-x

**Published:** 2017-08-31

**Authors:** Elisa Torre

**Affiliations:** Nobil Bio Ricerche srl, Via Valcastellana, 26, 14037 Portacomaro, AT Italy

**Keywords:** Anti-inflammation, Antioxidant, Bone disease, Pathway, Polyphenols

## Abstract

For millennia, in the different cultures all over the world, plants have been extensively used as a source of therapeutic agents with wide-ranging medicinal applications, thus becoming part of a rational clinical and pharmacological investigation over the years. As bioactive molecules, plant-derived polyphenols have been demonstrated to exert many effects on human health by acting on different biological systems, thus their therapeutic potential would represent a novel approach on which natural product-based drug discovery and development could be based in the future. Many reports have provided evidence for the benefits derived from the dietary supplementation of polyphenols in the prevention and treatment of osteoporosis. Polyphenols are able to protect the bone, thanks to their antioxidant properties, as well as their anti-inflammatory actions by involving diverse signaling pathways, thus leading to bone anabolic effects and decreased bone resorption. This review is meant to summarize the research works performed so far, by elucidating the molecular mechanisms of action of polyphenols in a bone regeneration context, aiming at a better understanding of a possible application in the development of medical devices for bone tissue regeneration.

## Introduction

Bone loss is a consequence of changes that occur in the bone cell activity during bone remodeling, which causes an imbalance between bone resorption and formation and leads to bone disorders, such as osteoporosis and increased fracture risk (Manolagas 2000). During normal physiological remodeling, in which the mature skeleton undergoes continuous regeneration, bone formation follows resorption in a “coupled” mechanism controlled by varied molecular factors. Unequal effects of these factors could lead to the imbalance responsible for the decrease of bone mass, in which extension of the working lifespan of the osteoclast coexists with shortening of the working lifespan of the osteoblast (Khosla et al. 2012). Various cell types are involved in the remodeling process, each type playing different roles in bone turnover: osteoblasts supporting bone formation, osteoclasts involved in bone resorption and osteocytes playing a central role by acting as master signal sensors, integrators and transducers in the remodeling compartment, with their multiple endocrine functions implicated in the regulation of both osteoclast and osteoblast activities (Bonewald 2011).

Polyphenols are phytochemicals commonly found in the plant kingdom, whose multiple biological effects have been reported to be protective against chronic diseases, including neurodegenerative and cardiovascular disease, cancer and osteoporosis (Scalbert et al. 2005). The beneficial actions of phenolic compounds are mainly due to their antioxidant properties, since they can act as scavengers of reactive oxygen species (ROS) (Procházková et al. 2011), but also to their interaction with intracellular signaling cascades such as phosphatidylinositol-4,5-bisphosphate 3-kinase (PI3K), protein kinase B (PKB)/Akt, tyrosine kinases, protein kinase C (PKC) and mitogen-activated protein kinases (MAPKs) (Nomura et al. 2001; Lin 2002; Kern et al. 2007; Larsen et al. 2010), that lead to anti-inflammatory, chemopreventive and chemotherapeutic activities.

**Fig. 1:**
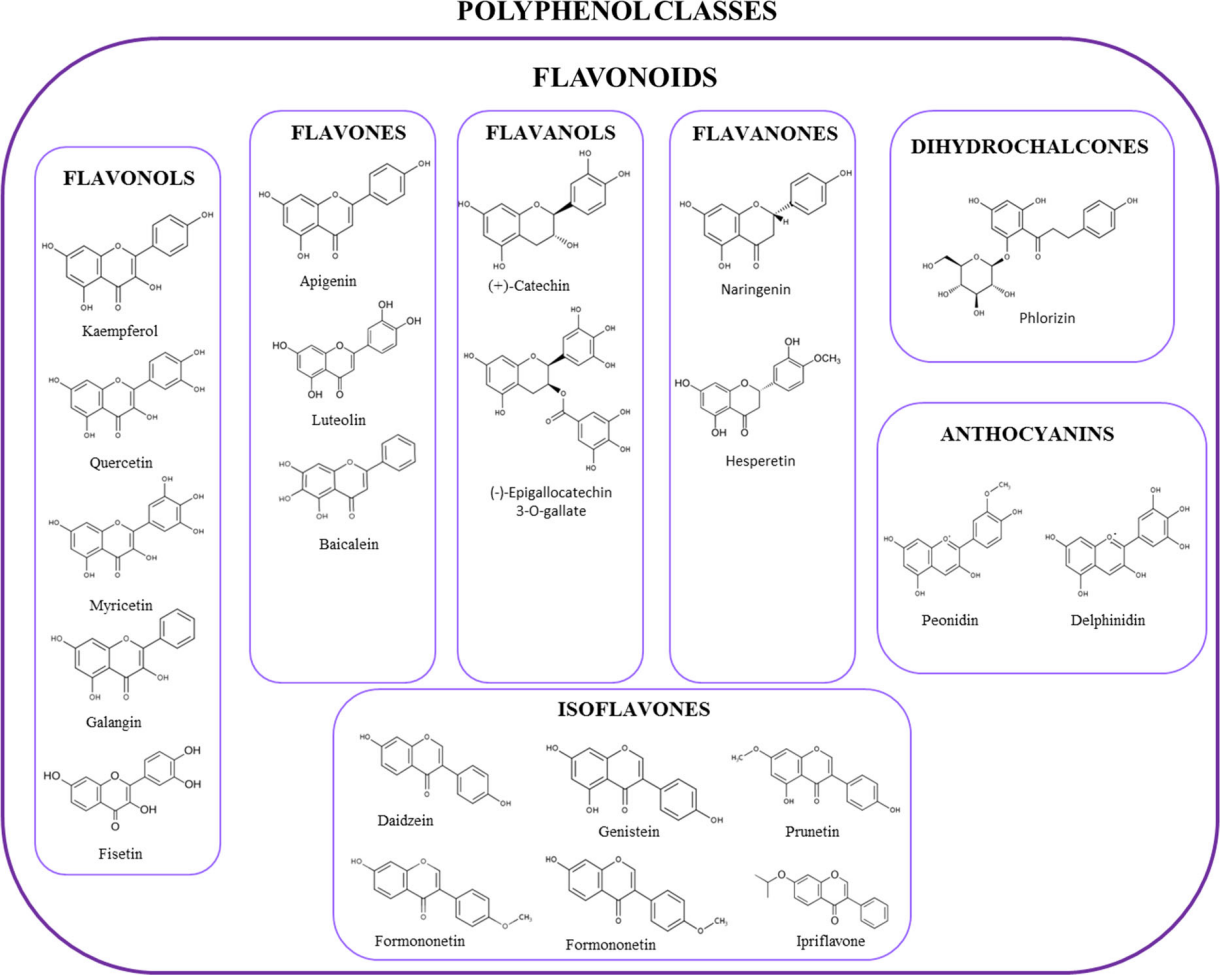
Polyphenol classification. Principal classes of polyphenols and their relative most effective compounds (Rothwell et al. 2013)

**Fig. 2:**
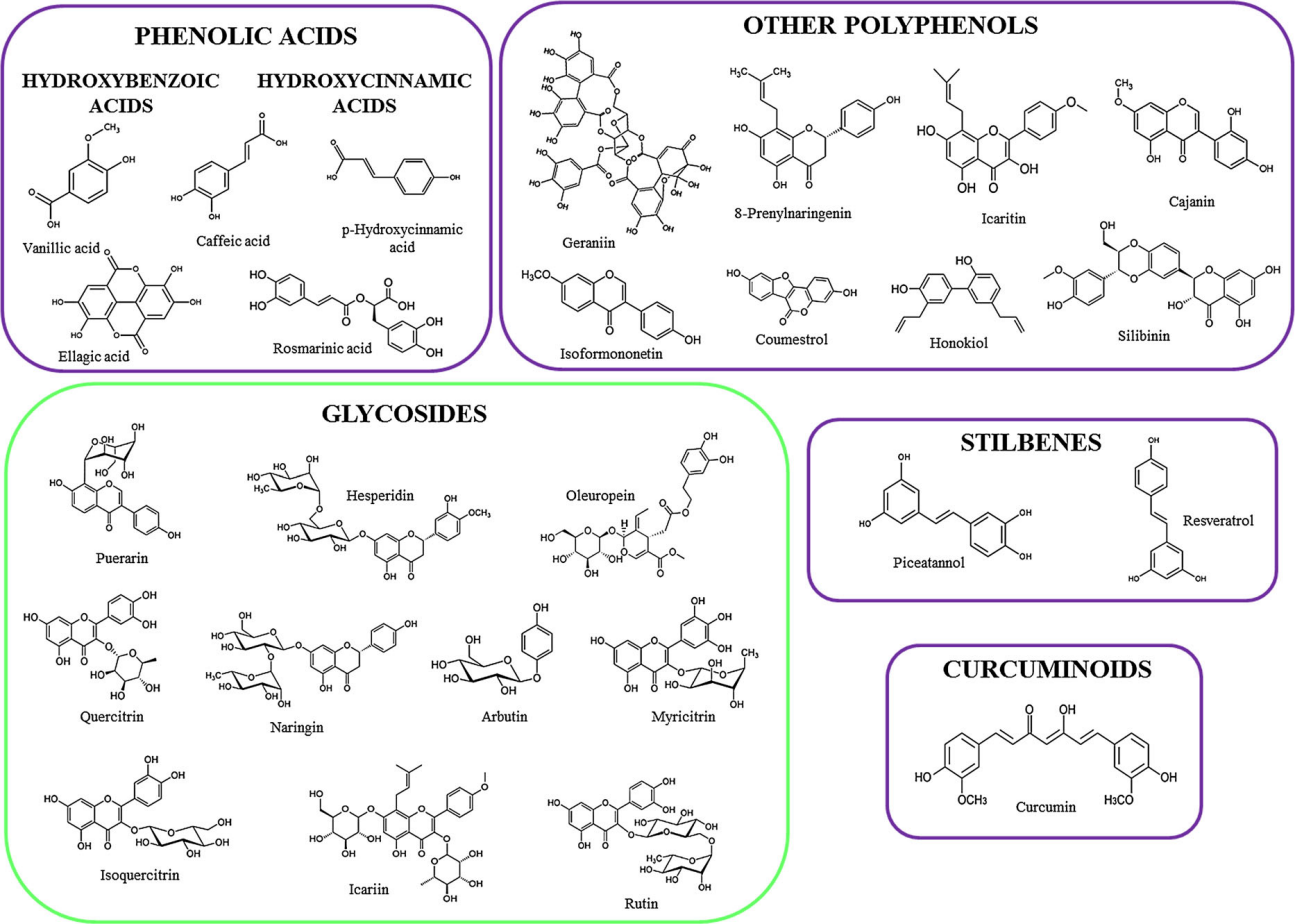
Polyphenol classification. Principal classes of polyphenols and their relative most effective compounds (Rothwell et al. 2013)

Depending on the number of phenol rings they contain and on the radicals bound to them, polyphenols can be divided into different groups: phenolic acids, flavonoids, stilbenes, tannins, coumarins and lignans (Fig. 1–2) (D’Archivio et al. 2007). Given that the chemical structure of a compound is related to its biological/toxicological activity (McKinney et al. 2000), polyphenols mode of action can be different, depending also on which concentration and on which biological system is used (Khlebnikov et al. 2007). However, it is quite difficult to quantitatively establish the benefits afforded by polyphenols, because of the limited understanding of their bioavailability; generally, the small intestine can absorb polyphenols in the form of aglycones, but many of them in their native form are esters, glycosides or polymers that cannot be absorbed by the gut barrier (Crozier et al. 2009). Hence, these compounds must be metabolized by intestinal enzymes or the gut microflora (D’Archivio et al. 2007). Many studies have found correlations between intake of polyphenols and bone health (Henrotin et al. 2011; Shen et al. 2011; Rao et al. 2012; Welch and Hardcastle 2014), mainly due to their antioxidant properties, because oxidative stress plays an important role in the pathogenesis of osteoporosis with its promotion of an increase in bone resorption linked to direct/indirect actions on the differentiation and activity of osteoclasts (Callaway and Jiang 2015). Besides their scavenging properties, polyphenols can influence bone metabolism through downregulation of inflammatory mediators (Bodet et al. 2007), such as cytokines, primarily implicated in sustaining osteoclast differentiation and activity (Palmqvist et al. 2002; Park and Pillinger 2007; Yao et al. 2008), thus contributing to a reduction in bone resorption. Another important aspect to be taken into account is the bone anabolic effect exerted by polyphenols, shown by many experimental evidence which highlighted how it is promoted by effects on the osteoblast involving different signaling pathways such as Wnt/β-catenin (Chen et al. 2010), insulin-like growth factor (IGF1) (Bu et al. 2009), bone morphogenetic proteins (BMPs) (Trzeciakiewicz et al. 2010a), Runt-related transcription factor 2 (Runx2) (Byun et al. 2014) and Osterix (Osx) (Santiago-Mora et al. 2011). Furthermore, because of a structural similarity to mammalian estrogens, some polyphenols such as isoflavones are also called phytoestrogens and are able to bind to estrogen receptors (ERs) α and β, thus acting as hormone analogs with different agonistic or antagonistic actions, depending on the tissue (Patisaul and Jefferson 2011).

As can be seen, their involvement in pathways that can cross-talk to other multiple transduction signals makes phenolic compounds a promising natural source to be employed in the development of plant-based therapeutics, with a wide application ranging from bone diseases, to cancers (Chen et al. 2014b), atherosclerosis (Loke et al. 2010), obesity (Tucakovic et al. 2015), diabetes (Dragan et al. 2015) and neurodegenerative disorders (Ebrahimi and Schluesener 2012). However, despite the renewed scientific interest in drug discovery from natural sources and the increasing demand in today’s society for natural compounds (Chang and Jeong 2015; Rajesh et al. 2015; Farha and Brown 2016), still insufficient data are available to establish the real value of these compounds in the context of public health or clinical practice. Hence, it will be necessary a deeper study of the molecular mechanisms underlying polyphenol modes of action, with an even more detailed knowledge of the interaction of phenolic compounds with their molecular targets, to better clarify their pharmacological activity and, subsequently, to properly optimize medicinal chemistry approaches and more appropriate clinical trial designs, as well as the development of advanced biomaterials and improved tissue-engineering approaches.

Here, we discuss the molecular mechanisms involved in the anabolic effects induced by polyphenols, highlighting the signaling pathways shared between the diverse classes of phenolic compounds, in terms of a better understanding of an even greater application of these natural compounds in the bone tissue regeneration field.

## Estrogen signaling pathway

Among sexual steroids, estrogens are the main female hormones that, in addition to their action in the development and maintenance of normal sexual and reproductive functions, play important roles in the control of different biological processes, with effects on the cardiovascular, musculoskeletal, immune and central nervous system (Gustafsson 2003).

The biological effects of estrogens are mediated through two distinct intracellular receptor forms, ERα and ERβ, each encoded by different genes located on different chromosomes (Gosden et al. 1986; Kousteni et al. 2003).

**Fig. 3:**
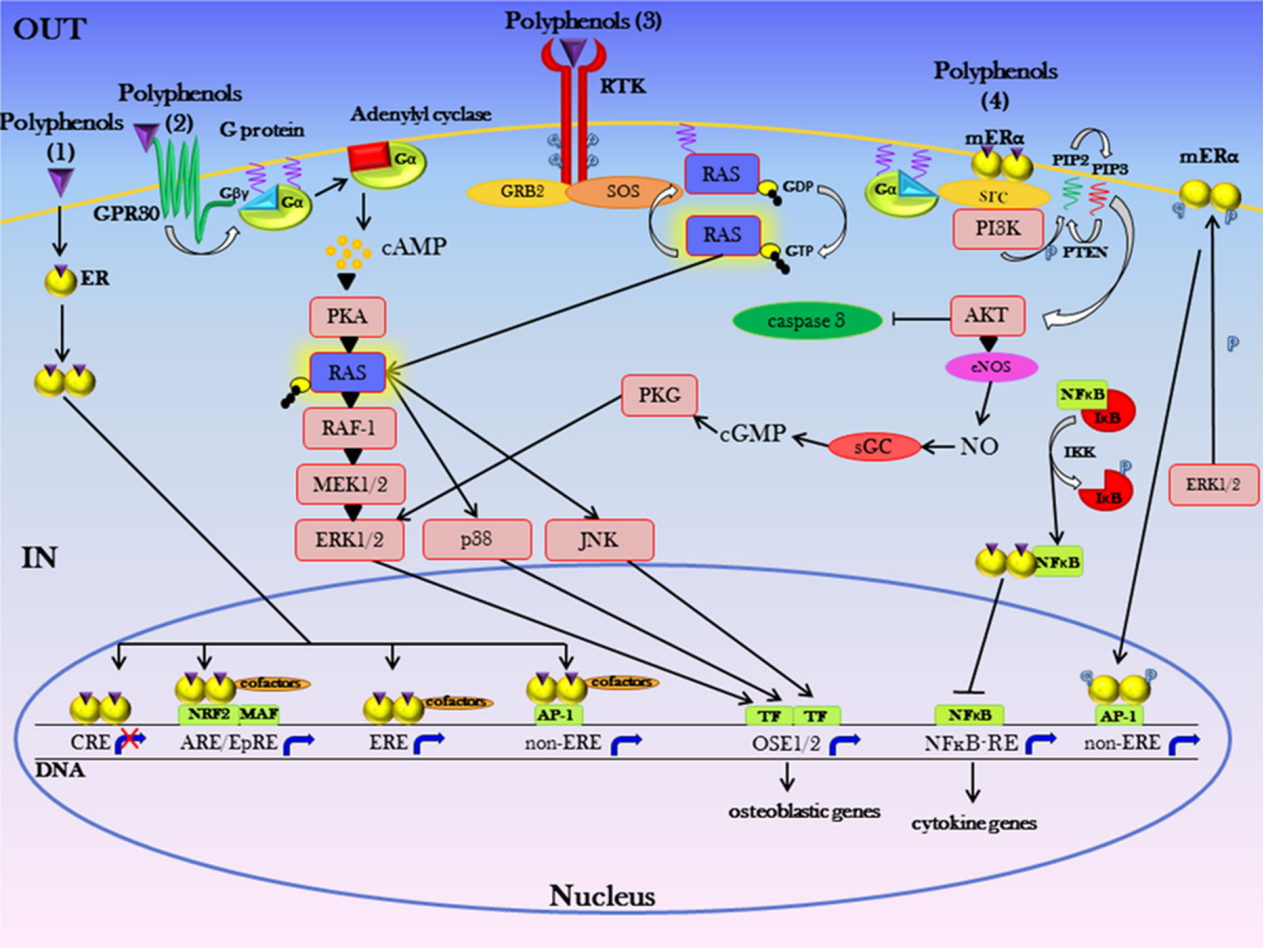
Influence of polyphenols on bone metabolism through estrogen signaling. Binding of phenolic compounds to ERs leads to activation of the canonical and noncanonical estrogen pathways, with a crosstalk with MAPKs and PI3K/Akt signaling. *GRB2* growth factor receptor-bound protein 2, *SOS* son of sevenless, *RTK* receptor tyrosine kinase, *GDP* guanosine diphosphate, *OSE1/2* osteoblast-specific element ½, *MAF* MAF protein, *G*_*α*_ G protein α subunit, *G*_*βγ*_ G protein βγ subunits. (1) Resveratrol, curcumin, daidzein, genistein, kaempferol, puerarin, coumestrol, apigenin, quercetin. (2) Vanillic acid, icariin, prunetin, resveratrol, daidzein, genistein, quercetin, kaempferol. (3) Daidzein, genistein, resveratrol, icariin, quercetin, kaempferol. (4) Resveratrol, genistein, daidzein, quercetin, rutin

Polyphenolic non-steroidal plant compounds with estrogen-like biological activity, estrogen receptor binding, ER-transactivation and estrogen dependent target gene expression are classified as phytoestrogens (Cos and Apers 2003) or selective estrogen receptor modulators (SERMs) and, as such, they can modulate the estrogen-dependent pathway by acting as partial agonists and/or antagonists of the ER in a tissue type and ligand concentration-dependent manner (Moutsatsou 2007). By activating the estrogen pathway, polyphenols are thus molecules able to regulate the expression of genes which, in bone, are responsible for the maintenance of bone mass, through a proper balancing between bone resorption and bone formation (Cauley 2015) (Fig. 3).

Based on their chemical structure, they can be classified into four main groups, which include isoflavonoids, flavonoids, stilbenes and lignans. Because of the structural similarity between phytoestrogens and 17β-estradiol (E2), based on the phenolic ring required for binding to the ER, as well as the presence of two hydroxyl groups (Harris et al. 2005), phytoestrogens exert their estrogenic activities by binding to ERs (Morito et al. 2001), thus activating the ER-dependent gene transcription, with a higher relative binding affinity for ERβ than ERα (Kuiper et al. 1998; Casanova et al. 1999). This relative selective binding of phytoestrogens to ERβ indicates that they may produce different effects from those exerted by estrogens, since estrogens bind to both ERα and ERβ with almost the same affinity (Morito et al. 2001), thus triggering distinct ER-mediated transcriptional events. On the other hand, some polyphenols, such as 8-prenylnaringenin (8-isoprene-4,5-7-hydroxy flavanone, isolated from the female flowers of *Humulus lupulus*), have been shown to preferentially bind to ERα than to ERβ and to promote osteoblast differentiation and inhibition of bone resorption with a stronger effect, compared to genistein and daidzein, at a dose of 10 μM (Luo et al. 2014).

Besides the predominant effects of ERβ, a wide range of structural forms of the ligand-receptor complex occur in generating a wider range of action for phytoestrogens, thus recruiting different co-activator or co-repressor proteins (Routledge et al. 2000). Furthermore, the potential hormonal effects of phytoestrogens on osteoblasts is pertinent with the different expression of the ER forms during the osteoblast differentiation stages, since ERβ is found to be greatly expressed during bone mineralization (Arts and Kuiper 1997). Binding of the ER with different compounds induces different conformational changes in the receptor.

Classical binding of estrogens to the ER in the cytosol, leads to a conformational change within the ER that promotes homodimerization, recruitment of the ER to the promoter region of transcription start sites, high affinity binding to specific cis-acting enhancers DNA response elements (ERE) located within the regulatory regions of target genes and recruitment of coactivators that stimulate gene transcription (O’Lone et al. 2004). In the case of genes whose promoters don’t harbor EREs, ligand-bound ER can interact with transcription factor complexes like activator protein-1 (AP1) or specificity protein-1 (SP1), that tether the ER to the promoter, a process referred to as “non-ERE” or “transcription factor cross-talk” (Gustafsson 2003). Thus, phytoestrogens can act as pure agonists, as partial agonists or as pure antagonists. Different results, in literature, are given about agonistic or antagonistic activities of polyphenols, in fact coumestrol, apigenin, daidzein and genistein exhibit a strong agonistic activity for ERs at concentrations ranging from 10 μM to 10 nM, while resveratrol, naringenin (a flavonoid found in *Citrus medica*), kaempferol and quercetin have been shown to have weak or even antagonistic activity for both ERα and ERβ (Miodini et al. 1999; Harris et al. 2005; Tang et al. 2008b). Conversely, other authors found that quercetin mediates ERE-dependent transactivation with effects on stimulation of osteoblastic proliferation (Van Der Woude et al. 2005; Veprik et al. 2012).

Other phytoestrogens, following binding to the ER, have been observed to negatively target bone resorption through the classical ERE-mediated ligand-dependent pathway (Fig. 3). In fact, a possible interaction for quercetin and kaempferol with the ER, at concentrations of 0.1–10 μM, has been speculated on the basis of their inhibitory effects on bone resorption—although the estrogenic potency of kaempferol is greater than quercetin—significantly reversed by the use of the ER antagonist ICI 182780 (Wattel et al. 2003) and confirmed in a subsequent report showing, furthermore, that quercetin is able to act as selective ER modulator by upregulating ERβ and downregulating ERα expression (Rassi et al. 2005). Similarly, inhibition of osteoclastic bone resorption in rats and, conversely, stimulation of osteoblastic bone formation following a diet enriched with phlorizin (a flavonoid exclusively found in apple) 2.0 × 10^−4^ mol/day and rutin 4.1 × 10^−3^ mol/kg have been postulated to be mediated through the ER (Horcajada-Molteni et al. 2000; Puel et al. 2005).

Apart from acting through EREs, phytoestrogens have been shown to interact, through the ERs, with other response elements, such as the antioxidant response element/electrophile responsive element (ARE/EpRE), thus inducing the transcription of the phase II detoxification enzymes (Fig. 3). Evidence for phytoestrogen modulation of ARE-regulated transcription is provided by Veprik et al., that report the involvement of the nuclear factor E2-related factor 2 (Nrf2)/ARE transcription system in the activation of estrogen signaling in two osteoblast-like cell lines (Veprik et al. 2012), while cyclic adenosine monophosphate (cAMP) response elements (CREs) have been shown to be targeted by soy isoflavones, which suppress CRE-mediated transcriptional activity through ERs and mRNA expression of genes that contain CRE/CRE-like elements in their promoter in osteoblastic cells (Tang et al. 2011).

Phytoestrogens not only target the classical ER pathway, but also the rapid non-genomic signaling, in a ligand-dependent or independent manner (Fig. 3). The “nongenomic” action differs from the genomic one, since it involves a series of rapid events deriving from the interaction between cell-surface ER forms that are linked to intracellular signal transduction proteins, such as the G protein-coupled receptor 30 (GPR30). These non-genomic events may be mediated by diverse main signaling cascades: phospholipase C (PLC)/PKC, Ras/Raf/MAPK, PI3K/AKT and cAMP/protein kinase A (PKA) (Björnström and Sjöberg 2005).

Vanillic acid (VA), isolated from *Sambucus williamsii*, for example, differs from other phytoestrogens like genistein, because it does not bind to either ERα or ERβ, nor induces ERE-dependent transcription. In fact, VA has been shown to up-regulate the expression of osteoblastic differentiation markers, such as Runx2, osteocalcin (OCN) and osteoprotegerin (OPG), by activating the rapid non-genomic ER pathway at concentrations of 0.01 μM and 0.1 nM, through phosphorylation of MEK1/2, ERK1/2 and ERα (Xiao et al. 2014b). Also ipriflavone (7-isopropoxyisoflavon, isolated from *Medicago sativa*) has been shown not to bind to the ER, but to a unique steroid receptor superfamily binding site in the nucleus of pre-osteoblastic cells and not to induce ERE-dependent gene transcription (Petilli et al. 1995). Furthermore, icariin, the principal flavonoid glycoside found in *Herba Epimedii*, also acts like a phytoestrogen through the non-classical ER-dependent pathway (Xiao et al. 2014a), because its effects on osteoblast proliferation, differentiation and mineralization, at doses ranging from 5 to 40 μM, are reached by activating AP-1 through the up-regulation of c-fos and c-jun via activation of extracellular signal–regulated kinase (ERK) and c-Jun N-terminal kinase (JNK) pathways (Song et al. 2013; Wu et al. 2015b). Thus, icariin could have therapeutic effects on osteoporosis (Zhang et al. 2009), by enhancing osteoblastic differentiation and suppressing osteoclastic differentiation (Chen et al. 2007; Huang et al. 2007; Hsieh et al. 2011).

G protein-coupled estrogen receptor 1 (GPER), also known as GPR30, is a member of the 7-transmembrane G protein-coupled receptor (GPCR) family, capable of mediating both transcriptional and nongenomic events in response to estrogen (Prossnitz et al. 2008). An example of a positively acting polyphenol on bone metabolism, through binding to the GPR30, is given by prunetin isoflavone (found in red clover and fruit of *Prunus avium*), which, at 0.01 μM, selectively binds to the GPR30, thus stimulating osteoblast proliferation and differentiation through the production of cAMP and through activating ERK/MAPK, as well as leading to expression of Runx2 in osteoblasts (Khan et al. 2015) (Fig. 3).

Given the evidence that the ER is expressed by mesenchymal stem cells (MSCs), osteoblasts and osteoclasts (Vidal et al. 1999; Windahl et al. 2000), it is clear that estrogen and estrogenic compounds exert pleiotropic effects on bone metabolism on the basis of which cell type they target.

First of all, phytoestrogens are capable of influencing MSCs, by enhancing osteogenic differentiation, while suppressing the adipogenic one via a nongenomic mechanism ER-mediated (Li et al. 2005). In this context, supplementation of 1 μM genistein has been reported to increase osteogenesis in human bone marrow stromal cells (hBMSCs) at day 18 of incubation, by acting on gene expression markers, such as Runx2 and alkaline phosphatase liver/bone/kidney (ALPL), involved in the early stages of differentiation of human primary MSCs (Heim et al. 2004). Ability of isoflavones to suppress adipogenic differentiation of adipose tissue-derived (AD) MSCs has also been investigated in the contest of Wnt/β-catenin signaling using an estrogen antagonist. The results show that these phytoestrogens, at 0.01–100 μM, do inhibit AD-MSCs differentiation in mature adipocytes through a stimulation of Wnt signaling mediated by both non-genomic and genomic ER-dependent pathways (Kim et al. 2010). These opposite effects on osteogenic and adipogenic differentiation are likely due to a different expression of the ER subtypes in the MSCs during the developmental stages, implying cell-specific differences in the estrogenic sensitivity. Indeed, all ERs already present in MSCs are up-regulated during osteogenesis, with the β5 splice variant strongly expressed and, except for ERα, downregulated during adipogenesis (Heim et al. 2004). Effects on MSCs proliferation have also been seen following treatment with resveratrol, which has been shown, at 1 μM, to directly stimulate cell proliferation, osteoblastic differentiation and osteogenic gene expression through induction of ER signaling and MAPK activation, with involvement of ERK1/2 and p38, playing a positive and a negative role on cell proliferation and osteoblast differentiation, respectively (Dai et al. 2007) (Fig. 3).

The role of polyphenols in bone anabolism is further supported by in vitro studies investigating the effects of isoflavones on osteoblast activity, showing increased protein synthesis, DNA content and alkaline phosphatase activity (Yamaguchi and Gao 1997); given that the presence of E2 caused a significant increase in protein content and alkaline phosphatase activity and that the anti-estrogen tamoxifen blocked the effects, the mechanism proposed by the authors partly involves the estrogen pathway (Yamaguchi and Gao 1997; Sugimoto and Yamaguchi 2000a, b; Yamaguchi and Sugimoto 2000). A study from Guo et al. (2012a) showed that kaempferol, at 50 μM, is able to stimulate osteogenic differentiation of cultured osteoblasts by the activation of ERα via a classical ER signaling pathway, while quercetin, at low concentrations (1–10 μM) and curcumin and resveratrol at 2.5 and 10 μM, respectively, have been shown to stimulate cell proliferation (Van Der Woude et al. 2005; Veprik et al. 2012). Isoflavones such as daidzein and genistein, have been shown to stimulate osteoblast differentiation through enhancing Runx2 expression levels and bone morphogenetic protein (BMP)-2 signaling with a mechanism involving the ER (Jia et al. 2003; Dai et al. 2013; Hinenoya 2013). Increased Runx2 expression, the master osteogenic transcription factor playing a major role in osteoblast maturation (Spilmont et al. 2013), is thus an obvious consequence of stimulation of osteoblastogenesis: ellagic acid (EA), for example, increases Runx2 expression by acting as a prebiotic in the intestine (Li et al. 2015b), thus contributing to the enhancement of calcium (Ca) absorption (Roberfroid et al. 2010) and pathways involving the ER (Papoutsi et al. 2005, 2008).

Osteoblastic activity has also been demonstrated to be stimulated by the flavonoids quercetin and kaempferol at 50 μM, which significantly increased ALP activity through activating ERK downstream of the ER (Prouillet et al. 2004), with involvement of a nongenomic mechanism and by the isoflavone daidzein, which, at 1 nM, increased the amount of the transcription factor RUNX2, ALP expression and the mineralization rate of osteoblasts via ER-dependent pathways (De Wilde et al. 2004). A direct stimulatory action on bone mineralization via the ER has been recognized as a resveratrol-mediated effect, which dose-dependently (1 μM) increased ALP activity, suggesting an estrogen-like action for resveratrol (Mizutani et al. 1998). Increased ALP activity has also been observed following treatment with coumestrol (1 μM), genistein and daidzein, with a higher estrogenic activity for coumestrol than genistein and daidzein (Kanno et al. 2004a). In vitro studies with human and animal osteoblasts or osteoblast-like cell lines have also been carried out to explore the action of polyphenols on bone formation, showing suppressed proliferation and parallel stimulatory effects on the differentiation of osteoblasts (Choi et al. 2001; Yoshida et al. 2011).

Estrogen and genistein have been also demonstrated to upregulate OPG through a direct interaction with the ER in human osteoblast cultures (Hofbauer and Khosla 1999; Viereck et al. 2002) and to induce OPG transcription through a DNA-binding independent nuclear mechanism (Roforth et al. 2014) and, in support of these data, a progressive up-regulation in the OPG:receptor activator of nuclear factor kappa-B ligand (RANKL) ratio during the osteoblast differentiation establishes a role for genistein in the maintenance of bone homeostasis, with a major impact on the relative balance between osteoblast and osteoclast number. Polyphenols from *Drinaria fortunei* and *Pueraria mirifica*, have been demonstrated to stimulate osteoblast proliferation, to increase OPG/RANKL ratio and to upregulate the expression of osteoblast differentiation markers, such as collagen type 1 (Col1), OCN and ALP, in an ER-dependent manner (Wang et al. 2011; Sheu et al. 2012; Wong et al. 2013; Tiyasatkulkovit et al. 2014).

Given that estrogen can enhance osteoblast activity also through a nitric oxide (NO)-dependent mechanism (O’Shaughnessy et al. 2000), in which NO-cyclic guanosine monophosphate (cGMP) pathway stimulates osteoblast replication and ALP activity (Mancini et al. 2000), the role of this pathway has been investigated in mediating the action of genistein on growth and osteoblastic differentiation of MSCs cultures (Pan et al. 2005). The results show that genistein, at 1 μM, stimulates proliferation and osteoblastic differentiation of MSCs via activation of the ER-dependent NO-cGMP pathway, by upregulating Runx2 gene expression (Fig. 3). In contrast to these anabolic effects, genistein supplemented to rat models at high doses (1.85 × 10^−4^ mol/kg) causes adverse effects on bone cells (Li et al. 2012), probably via ER-independent mechanisms, whose results are in line with the reported genistein biphasic effect on the growth of breast cancer cells (Anderson et al. 1998). Stimulation of osteoblastic proliferation and differentiation via NO-cGMP signaling pathway has also been shown to be induced by resveratrol (1 μM), which structurally resembles E2 and, thus, mimics E2 activity (Song et al. 2006).

Shortening of osteoblast lifespan is one of the hallmarks that, together with increased osteoclast activity and survival, contributes to the emergence of the osteoporotic disease (Manolagas 2000). In this respect, phytoestrogens have been demonstrated to prolong osteoblast lifespan in an estrogen-like manner, through inhibiting tumor necrosis factor-α (TNF-α)-induced apoptosis (Suh et al. 2003).

The inhibitory effect of polyphenols on bone resorption has been widely studied, showing inhibition of osteoclast-like cell formation in mouse marrow cultures (Gao and Yamaguchi 1999a) and inhibitory effect on bone resorption induced by various bone-resorbing factors (Yamaguchi and Gao 1998), through an estrogen-like mechanism.

Concerning apoptosis, different experimental evidence emerge from literature indicating anti-resorbing actions of polyphenols directly exerted on mature osteoclasts and their progenitors, through a molecular mechanism ER-mediated that involves activation of caspase-8 and caspase-3 (Rassi et al. 2002, 2005). Furthermore, the activation of ER signaling by genistein increased transforming growth factor-β1 (TGF-β1) expression during osteogenesis, especially in the final stages of osteoblast maturation (Heim et al. 2004), thereby contributing to osteoclast apoptosis (Hughes et al. 1996; Houde et al. 2009).

The anti-resorbing properties of flavonols are mainly mediated by ERs, through the inhibition of receptor activator of nuclear factor kappa-B (RANK) protein, thus directly targeting osteoclast progenitors. In this respect, unlike estrogen which does not alter the expression of RANK, but acts on c-jun activity to regulate the differentiation potential of osteoclast progenitors (Shevde et al. 2000), rutin, at 0.01 μM, has been shown to down-regulate RANK protein (Rassi et al. 2005).

On the other hand, daidzein, genistein and coumestrol, at μM concentrations, exert anti-osteoclastogenic effects through an ER-dependent mechanism that regulates the expression of genes involved in osteoclast formation, such as c-fos and nuclear factor of activated T-cells 1 (NFATc1) (Karieb and Fox 2011).

Polyphenols exert their anti-resorbing action by also regulating inflammatory cytokines responsible for bone resorption and, subsequently, degenerative bone diseases (Fig. 3). In fact, a large number of cytokines have been shown to regulate osteoclast formation and function, thus influencing their ability to resorb bone. As the most potent cytokine stimulator of bone resorption in vitro (Lorenzo et al. 1987), interleukin (IL)-1 possesses the ability to directly (Jimi et al. 1999) and indirectly (Hofbauer et al. 1999) act on osteoclasts, thus contributing to the development of chronic inflammatory diseases such as periodontitis. Genistein, with its tyrosine kinase inhibitory activity, has been shown to regulate, at 10 μM, the IL-1β-induced activation of MAPKs in periodontal ligament cells (PDL) through a nongenomic mechanism involving the GPR30 (Luo et al. 2012). Conversely, Chen et al. (2002, 2003) described inhibition of IL-6 production and enhancement of OPG expression by genistein, as mediated through estrogen receptors and ERE-dependent pathways, thus regulating osteoclastogenesis. Direct stimulation of ERα and ERβ on osteoblasts by puerarin (daidzein 8-C-glycoside), the main isoflavone glycoside found in the Chinese herb radix of *Pueraria lobata* (Zhang et al. 2007), and genistein leads to increased OPG/RANKL ratio (Yamagishi et al. 2001) and decreased IL-6 levels, through an ERE-dependent direct genomic mechanism involving the ERβ and the ERα (Wang et al. 2014c). The work from Zhang et al. (2007) showed that these bone anabolic effects are mediated via activation of different signaling pathways cross-talking with the ER, such as the MAPKs and the PI3K/Akt (Zhang et al. 2007; Sheu et al. 2012; Wang et al. 2013b), following stimulation of the ERβ (Sheu et al. 2012) (Fig. 3). Soybean isoflavones can also inhibit secretion of TNF-α-induced IL-6 and prostaglandin E2 (PGE_2_) from osteoblastic cells, suggesting an anti-resorptive action of soy phytoestrogens (Suh et al. 2003). Furthermore, PGE_2_ production in osteoblasts is also inhibited by resveratrol, which suppresses proliferation of osteoclasts and stimulates mineralization (Morita et al. 1992).

Finally, given their antioxidant properties, polyphenols also counteract the deleterious effects of oxidative stress in osteoblastic cells, through different molecular mechanisms also involving the ER and the PI3K signaling pathways (Choi 2012).

Emerging evidence shows that a phytoestrogen-rich diet provides an array of potent biological activities. Results, however, are contradictory (Adlercreutz 2002; Adlercreutz and Heinonen 2004), in fact phytoestrogen hormonal activity depends on different factors, such as the metabolism, the route of administration, the dosage, the developmental stage, the chemical structure and the endogenous estrogenic status.

Furthermore, because the potency of phytoestrogens is much lower than estradiol, estrogenic effects of phytoestrogens on bone may be of minimal impact, or even antagonistic in the face of endogenous estrogen levels.

## Sirt1 signaling pathway

The sirtuins (silent information regulator 2—Sir2) are highly conserved nicotinamide adenine dinucleotide (NAD)-dependent enzymes that deacetylate residues of acetylated lysine, resulting in transcriptional silencing (Imai et al. 2000).

Sirtuin 1 (Sirt1) is a multifaceted class III histone deacetylase involved in a wide variety of cell processes, ranging from cancer to ageing, which has been conserved throughout evolution from yeast to human and is a crucial link between cell metabolism, longevity and stress response (Brooks and Gu 2009).

**Fig. 4:**
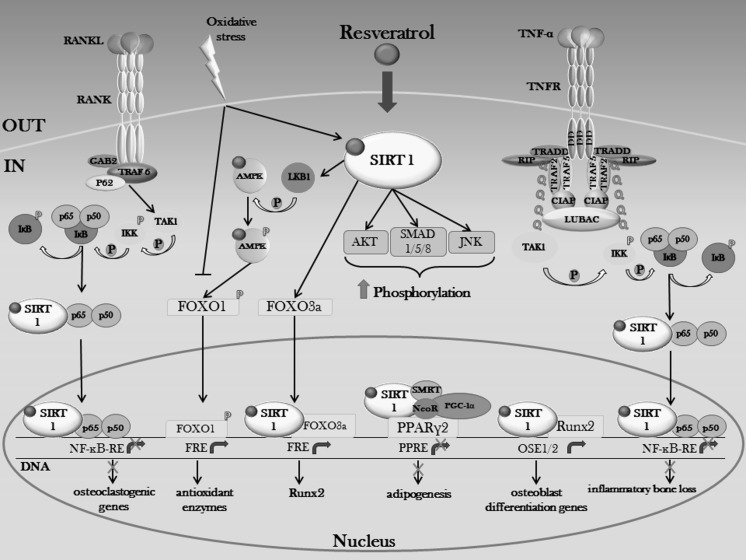
SIRT1-mediated bone anabolic effects of resveratrol. The ability of resveratrol to bind to SIRT1 and to subsequently increase its enzymatic activity provides a reply aimed at enhancing the osteoblast differentiation process, through the parallel decrease of adipogenesis. Impairment of osteoclast differentiation and function is also achieved through decreasing the DNA binding activity of NFκB, thus inhibiting RANKL and TNF-α-induced transcription of genes involved in osteoclastogenesis. *GAB2* GRB2-associated-binding protein 2, *TRADD* tumor necrosis factor receptor type 1-associated death domain, *RIP* receptor-interacting protein kinases, *CIAP* cellular inhibitor of apoptosis, *LUBAC* linear ubiquitination assembly complex, *PGC*-*1α* peroxisome proliferator-activated receptor gamma coactivator-1 alpha, *PPRE* PPAR response element, *FRE* FOXO response element, *Q* ubiquitin, *P* phosphorylation

Several studies (Schneider-Stock et al. 2012) have been shown evidence for a role of polyphenols in epigenetic modifications, by altering DNA methylation and histone modifications, thus leading to gene activation or silencing. One of the most potent activators of Sirt1 is resveratrol, because of its ability to bind to a special binding site in Sirt1, which induces a conformational change in the protein, resulting in an increased enzymatic activity (Howitz et al. 2003). Given the reciprocal relationship between osteogenesis and adipogenesis in MSCs, Sirt1 activation by resveratrol at 50 μM leads to decreased adipocyte differentiation and increased osteoblast differentiation (Bäckesjö et al. 2008). The mechanism by which resveratrol inhibits adipogenesis and mediates differentiation of MSCs to osteoblasts appears to involve, on one hand, a Sirt1-dependent indirect inhibition of peroxisome proliferator-activated receptor gamma (PPARγ), through the interaction of Sirt1 with nuclear receptor co-repressor (NCoR) (Shakibaei et al. 2012) and, on the other, the direct activation of Runx2 (Tseng et al. 2011) (Fig. 4). Given that Sirt1 has no inherent DNA binding ability, its effects on osteogenic differentiation are mediated through Runx2 transcription factor, by forming a Sirt1-Runx2 complex (Shakibaei et al. 2011), in which Sirt1 deacetylates Runx2, resulting in suppressed adipogenesis and activated osteogenesis (Shakibaei et al. 2012). These results were further confirmed by using immortalized human periodontal ligament cells, in which activation of Sirt1 by resveratrol, at 50 μM, increased mineralized nodule formation and upregulated the expression of mRNAs encoding osteoblastic markers (Lee et al. 2011b). Being resveratrol an agonist of Sirt1, its beneficial actions on osteoblastic differentiation are also achieved through production of Col1 and osteopontin (OPN). The precise mechanism of this induction is represented by activation of SIRT1 and diminished expression of pIκBα and nuclear factor kappa-light-chain-enhancer of activated B cells (NF-κB) subunit p65, thus promoting osteoblast differentiation (Feng et al. 2014) (Fig. 4). Moreover, resveratrol-mediated activation of Sirt1 enhanced phosphorylation of downstream kinases, reported to contribute to osteoblastic differentiation in bone cells and osteoblasts, such as PKB/Akt, Small Mother Against Decapentaplegic (SMAD)1/5/8, 5′-adenosine monophosphate protein kinase (AMPK) and MAPKs (Lee et al. 2011b). Resveratrol, at 5 μM, can also exert anti-osteoclastogenic effects via activating Sirt-1 pathway, in particular through inhibiting RANKL-induced NF-κB (Shakibaei et al. 2011), by reducing the levels of osteoclast activity markers, such as IL-6, TNF-α and tartrate-resistant acid phosphatase (TRAP)-5b and by contributing to maintaining a normal RANKL/OPG ratio (Zhao et al. 2015). Activation of Sirt1 pathway by resveratrol and the subsequent AMPK phosphorylation, repress the inflammatory responses mediated by the NF-κB/MAPK pathway, while the enhanced expression of antioxidant enzymes following activation of the Nrf2/antioxidant defense pathway leads to inducible nitric oxide synthase (iNOS) inhibition and, thus, to reduced nitrosative stress (Tamaki et al. 2014) (Fig. 4). Finally, resveratrol 2–50 μM also reverses the iron-overload-induced downregulation of Runx2, Col1 and OCN via Sirt1 activation, showing a potential in counteracting oxidative stress (Zhao et al. 2015).

Resveratrol also acts on bone architecture by promoting a proper bone remodeling, through reducing prostaglandin E1 (PGE1), prostaglandin D2 (PGD-2), prostaglandin F 2α (PGF2α) and basic fibroblast growth factor 2 (FGF-2)-stimulated OPG production, through a mechanism involving SIRT1 activation and inhibition of Akt and MAPKs signaling (Kuroyanagi et al. 2014a, b, c; Yamamoto et al. 2015).

## Mapks cascade

Transduction of extracellular signals to cellular responses is mediated by different information-processing circuits. These molecular circuits detect, amplify and integrate different external signals to generate molecular responses such as gene transcription and expression that translate to metabolic responses, which regulate cell proliferation, cell differentiation, metabolism, motility, survival and apoptosis (Zhang et al. 2002).

Mitogen-activated protein kinases are Ser/Thr protein kinases that transduce extracellular signals from membrane-bound activated tyrosine kinase receptors to the nucleus. The MAPKs pathway can be activated in most, if not all, of the vertebrate cells by a wide variety of receptor tyrosine kinases (TRKs), giving rise to multiple cross-talks with other signaling pathways thanks to the association with different scaffold proteins and to different docking motifs.

Several works (Ge et al. 2007; Ikeda et al. 2008; Matsuguchi et al. 2009; Thouverey and Caverzasio 2012; Lee et al. 2016) revealed that MAPKs are implicated in the regulation of bone mass, being mediators of osteoblast activity and osteoclast differentiation.

Activation of MAPKs signaling pathway by polyphenols has been demonstrated in different cellular systems, in a direct or indirect manner.

Their beneficial actions on bone metabolism are also achieved through molecular mechanisms targeting MAPKs pathway, which translate in regulation of osteoclast differentiation, bone resorption and promotion of osteoblast proliferation, differentiation and functions.

**Fig. 5:**
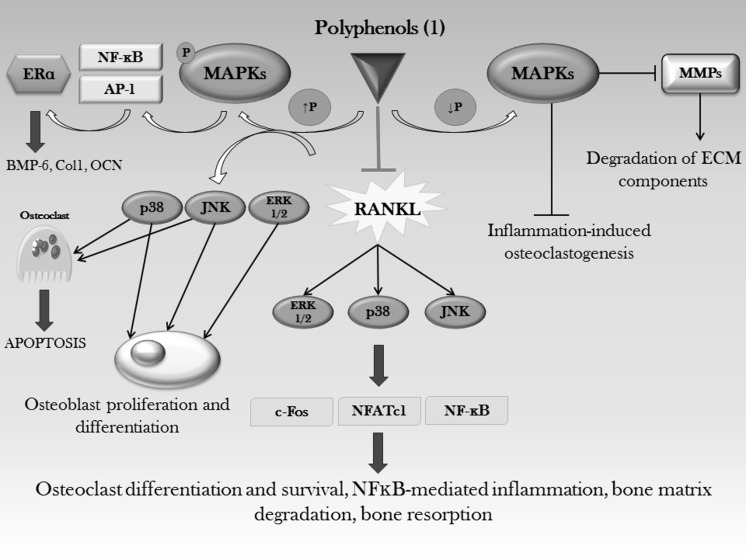
Polyphenol beneficial effects on bone diseases are mediated through actions on the MAPKs cascade. Different effects are obtained on the basis of MAPKs phosphorylation state, in fact polyphenols are able to either increase or to decrease phosphorylation, thus determining osteoblast proliferation and differentiation and inhibition of inflammation-induced osteoclastogenesis, respectively. (1) Geraniin, daidzein, genistein, quercetin, curcumin, caffeic acid, CADPE, furosin, coumestrol, EGCG, A-type proanthocyanidins, (2S)-2′-methoxykurarinone, icariin, apigenin, cajanin, isoformononetin, HCA, ugonin K, baicalein, quercitrin

Polyphenols have been shown to negatively act on genes involved in RANKL-induced osteoclast differentiation, such as NFATc1 (Zhao et al. 2010b), c-fos (Grigoriadis et al. 1994), NF-κB and AP-1, through regulating ERK1/2, p38 and JNK MAPKs expression and phosphorylation (Kim et al. 2006a; Pang et al. 2006; Murakami et al. 2007; Tsai et al. 2008; Kim et al. 2008b, 2009, 2011b; Huh et al. 2013; Léotoing et al. 2013; Nepal et al. 2013; Sakai et al. 2013; Heo et al. 2014; Lee et al. 2014b, 2015) (Fig. 5).

Cross-talking with other molecular signaling pathways is also a common fact, in fact the increased phosphorylation of MAPKs induced by polyphenols such as genistein, also induces ERα gene expression, which stimulates osteoblast differentiation and maturation, by increasing BMP-6, Col1 and OCN gene levels (Liao et al. 2014) (Fig. 5).

Osteoprotective effects (Lu et al. 2015) by geraniin (the main polyphenolic component of *Geranium thunbergii*) at nM concentrations (He et al. 2013), daidzein and genistein are exerted through inhibitory actions on osteoclastogenesis and osteoclast functions, by employing mechanisms mediated via suppression of ERK and inhibition of NF-κB activation, thus leading to impaired osteoclast formation and activity (Palacios et al. 2005; Xiao et al. 2015). Antagonizing action on osteoclast differentiation and, as a consequence, bone resorption is also reported by different works (Ozaki et al. 2000; Wattel et al. 2003; Bharti et al. 2004; Wattel et al. 2004; Woo et al. 2004; Yamaguchi et al. 2007; Siddiqui et al. 2011; Yamaguchi and Weitzmann 2011), in which quercetin and curcumin contribute to mitigate bone loss through a mechanism involving suppression of NF-κB and AP-1 (Wattel et al. 2004). Wu et al. (2012) found that treatment of ovariectomized mice with the phenolic compound caffeic acid 3,4-dihydroxy-phenethyl ester (CADPE) 3.5 × 10^−5^ mol/kg every 2 days inhibits NFATc1 expression, by targeting the MAPK/AP1 signaling pathway. Therefore, besides suppressing osteoclastogenesis, CADPE also impairs osteoclast activity through decreasing osteoclast-related marker genes, such as TRAP, cathepsin K and c-Src.

The same inhibitory effects are seen by following treatment with polyphenols, such as furosin, which targets the early stages of osteoclast differentiation through reducing the RANKL-induced phosphorylation of AP-1, p38 and JNK (Park et al. 2004), while coumestrol, at 10 μM, has been shown to have impact on late osteoclastic differentiation markers, such as matrix metalloproteinase (MMP)-9 and calcitonin receptor and the proposed mechanism includes decrease of ERK1/2 phosphorylation (Kanno et al. 2004b). Prevention of MMPs expression induced by *Porphyromonas gingivalis* in osteoclasts, has been shown to be also exerted by epigallocatechin gallate (EGCG) at 20 μM, maybe by blocking the MAPK signaling (Yun et al. 2004) (Fig. 5) and by A-type proanthocyanidins, which, at concentrations ranging from 10 to 50 mg/l, do inhibit osteoclast differentiation (Tanabe et al. 2011), lipopolysaccharide (LPS)-induced MMPs production and biofilm formation and modulate inflammatory responses to periodontopathogens, by inhibiting the phosphorylation of diverse signaling proteins, such as AP-1 and JNK (La et al. 2009a).

Several works report the anti-inflammatory effects of polyphenols, being exerted through targeting MAPKs pathway (Fig. 5): EGCG plays a role against inflammatory cytokines, which favor bone resorption, by inhibiting, at a dose of 30 μM, endothelin1 and platelet-derived growth factor BB-induced IL-6 synthesis, through diminishing the phosphorylation levels of MEK1/2 and Raf-1 at a point upstream of ERK1/2 MAPK (Tokuda et al. 2007a) and through downregulating the stress-activated protein kinase (SAPK)/JNK pathway (Takai et al. 2008); (2S)-2′-Methoxykurarinone, a compound isolated from the root of *Sophora flavescens*, inhibits, at the dose of 20 μM, IL-1-induced differentiation of osteoclasts through the inhibition of p38 and JNK phosphorylation (Kim et al. 2014), while icariin, at nM concentrations, decreases PGE_2_ production by suppressing activation of p38 and JNK pathways (Hsieh et al. 2011).

Triggering apoptosis in osteoclasts is also an event that contributes to modulating bone resorption, in fact EGCG has been shown to induce osteoclast apoptosis by decreasing RANKL-induced JNK activation (Lin et al. 2009; Lee et al. 2010a; Jin et al. 2011), while quercetin, at 50 μM, upregulates B-cell lymphoma 2 (Bcl-2)-associated X (Bax) protein expression, via a mechanism involving p38 and JNK MAPKs (Guo et al. 2012c) (Fig. 5).

Promotion of bone anabolism is also achieved through actions aimed at enhancing proliferation, differentiation and mineralization of osteoblasts.

In this respect, polyphenols like icariin and apigenin have shown induction of MSCs proliferation through modulating phosphorylation of ERK, p38 and JNK MAPKs (Qin et al. 2015; Zhang et al. 2015), while cajanin and isoformononetin, both found in *Butea monosperma* extract, at concentrations ranging from nM to pM, do stimulate osteoblast activity, proliferation and differentiation through activating MEK-ERK signaling pathways (Bhargavan et al. 2009).

p-Hydroxycinnamic acid (HCA), at concentrations of 0.01 and 0.1 μM, has been demonstrated to have anabolic effects on bone cells, which are carried out through stimulation of osteoblastic cell number, increase in calcium content, alkaline phosphatase activity and DNA content in vitro (Lai and Yamaguchi 2006a, b, 2008a, b).

Catechins are able to stimulate osteoblast differentiation and bone formation through regulating the ERK1/2 (Natsume et al. 2009), the p38 (Byun et al. 2014) and the SAPK/JNK (Tokuda et al. 2007b) MAPKs.

The pro-anabolic effects of HCA are also exerted through suppression of insulin-stimulated adipogenesis in pre-adipocytes and then favoring osteoblast differentiation, through a mechanism involving MAPK/ERK signaling (Yamaguchi et al. 2013).

Ugonin K (a flavonoid isolated from the roots of *Helminthostachys zeylanica*) and genistein are able to induce osteoblast differentiation through up-regulating the expression of Runx2 and Osx, via a mechanism involving phosphorylation of ERK1/2 and p38 MAPKs (Liao et al. 2007; Lee et al. 2011a).

Furthermore, genistein was reported to promote osteoblast differentiation and mineralization in vitro through suppressing DNA-binding of NF-κB (Kim et al. 2005) and LPS-induced activation of NF-κB (Hämäläinen et al. 2007), although Yamaguchi and Weitzmann (2009a) found a significant increase in NF-κB activity and even no antagonistic effects on TNF-α-induced NF-κB promoter activity, suggesting that the observed differentiation effect on osteoblastic cells is not mediated through suppressing NF-κB. Baicalein at 10 μM has been demonstrated to control expression of specific osteoblastic genes, such as OCN, OPN and Col1 through regulating the activation of NF-κB and AP-1 transcription factors via MAPK signaling at the early and the late stages of osteoblast differentiation, respectively (Kim et al. 2008a).

Hydroxyflavones have been displayed ability to stimulate osteoblastic differentiation and in increasing ALP activity via ERK and JNK signaling activation (Lai et al. 2014).

Polyphenols also favor osteogenesis through acting on mechanisms of regulation, such as phosphatases, which control different signaling pathways. For example, catechin, at 1 μM, has been seen to stimulate protein phosphatase 2A (PP2A), which regulates ERK activity by dephosphorylating it (Wei et al. 2011).

Quercetin, at 10 μM, promotes osteoblast differentiation through stimulating the expression of TGF-β1, BMP-2 and Runx2, via activation of ERK1/2, p38 and JNK MAPKs (Li et al. 2015a) (Fig. 5). However, quercetin is a flavonoid whose effects are both concentration and cell type dependent. Thus, different and, sometimes, opposite effects can be seen depending on which experimental model is used (Zhou et al. 2015): induction of apoptosis (Son et al. 2006; Nam et al. 2008), through activation of ERK-induced caspases (Nam et al. 2008) and JNK-mediated mechanisms (Son et al. 2008); inhibition of proliferation, differentiation, migration and mineralization in vitro (Notoya et al. 2004; Nam et al. 2008; Yamaguchi and Weitzmann 2011); increased alkaline phosphatase (Prouillet et al. 2004) and other marker proteins of osteoblastic cells (Kim et al. 2006b); stimulation of bone calcification (Yamaguchi et al. 2007).

Furthermore, quercetin, at doses ranging from 5 to 20 μM, is less efficient than kaempferol, at the same concentrations, in regulating the RANKL-induced expression of c-fos, which is required for osteoclast differentiation (Pang et al. 2006), while opposite results show an osteoblast protection effect against TNF-α-induced apoptotic cell death and prevention of H_2_O_2_-related cell death (Nam et al. 2008) through an ERK-dependent mechanism. Besides stimulation of proliferation and osteogenic differentiation, quercetin and quercitrin also exert angiogenetic effects, partially mediated through ERK and p38 MAPKs (Choi 2012; Zhou et al. 2015). Similarly to quercetin, curcumin has been demonstrated to dose-dependently induce apoptosis (12.5–25 μM) and necrosis (50–200 μM) in osteoblasts (Chan et al. 2006) by increasing reactive oxygen species (ROS) and decreasing adenosine triphosphate (ATP) levels, while on the other hand it has been demonstrated to decrease the rate of apoptosis dexamethasone-induced, by up-regulating the expression level of ERK1/2 (Chen et al. 2016).

Although HCAs have been shown to counteract some deleterious effects on skeletal system, caffeic acid may also impair bone mechanical properties (Folwarczna et al. 2009; Zych et al. 2010), showing how phenolic acids differently regulate bone. Concerning these different results, a deeper investigation on rats treated with phenolic acids led to dose-dependent differential effects: high doses (2.77 × 10^−4^ mol/kg/day caffeic acid, 2.82 × 10^−4^ mol/kg/day chlorogenic acid) do favor bone anabolism, while low doses (2.77 × 10^−5^ mol/kg/day caffeic acid) do impair it (Folwarczna et al. 2015). Possible mechanisms of action have been speculated, based on general findings that identify polyphenol-promoted bone growth via p38 MAPK/β-catenin Wnt canonical signaling (Chen et al. 2010).

The protective antioxidant properties of polyphenols have been shown to be mediated through increased phosphorylation of ERK1/2 and pNrf2, superoxide dismutase 1 (SOD-1) and heme oxygenase 1 (HO-1) protein levels (Braun et al. 2011; Choi 2012). Quercitrin glycoside counteracts the deleterious effects of oxidative stress in osteoblastic cells, through different molecular mechanisms also involving p38 pathway (Choi 2012).

## Inflammatory response pathway

Inflammation is the process by which the immune system responds to infections and injuries, thus enabling the removal of harmful stimuli and the healing of damaged tissues, aimed at restoring the host homeostasis. It is a complex series of events that includes its initiation, regulation and resolution, with a variety of forms triggered by different stimuli and numerous cross-talking molecular mechanisms (Abbas et al. 2012).

Several studies have investigated the anti-inflammatory and immunomodulatory activity of polyphenols, showing their interaction with a wide spectrum of molecular targets central to the inflammatory signaling, thereby exerting inhibitory effects on the production of inflammatory mediators and antioxidant detoxifying actions (González-Gallego et al. 2010).

**Fig. 6:**
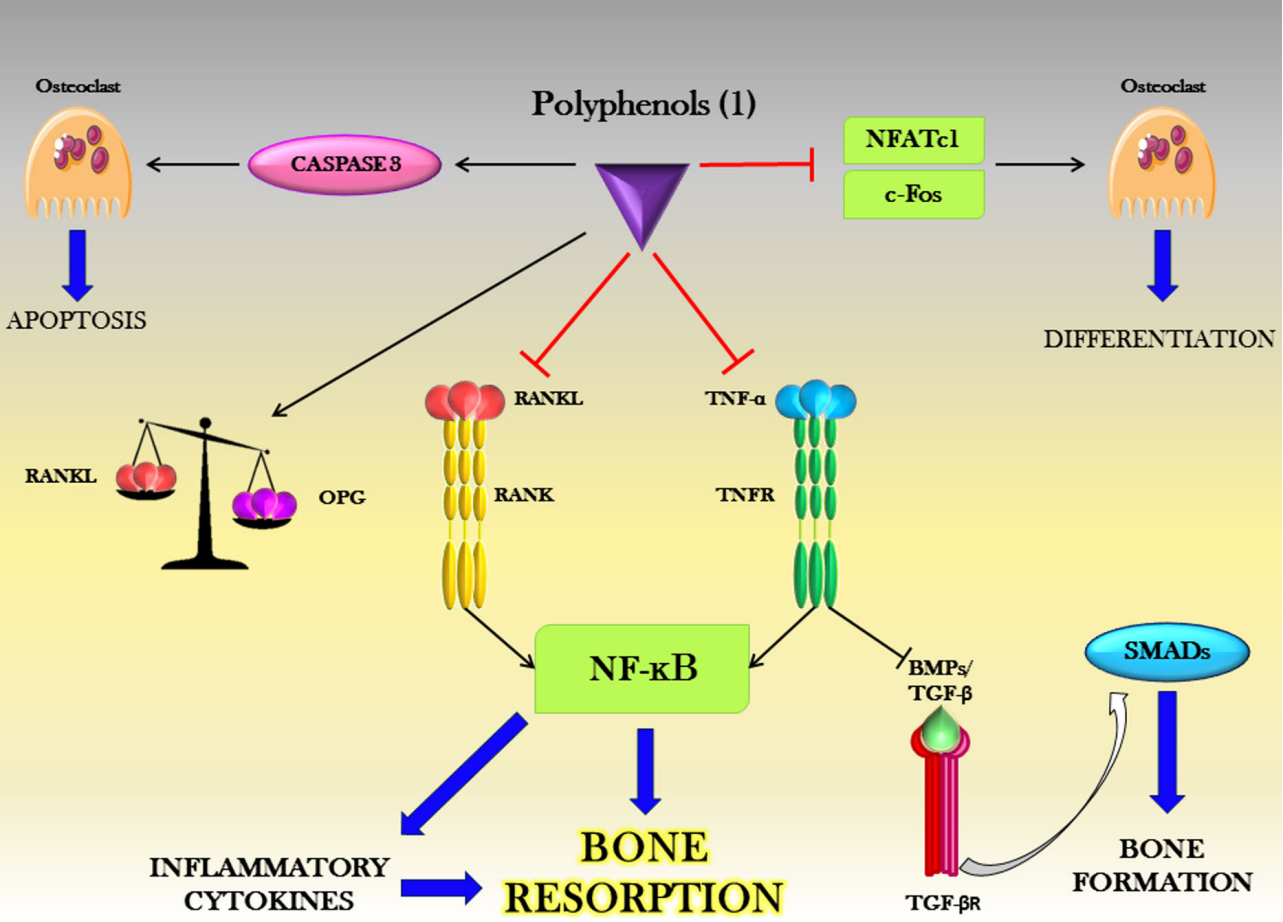
Anti-inflammatory properties of polyphenols in controlling bone resorption. Inflammation-induced activation of NF-κB is inhibited by polyphenols, which are effective in triggering osteoclast apoptosis and inhibiting osteoclast differentiation. Thus, they play a role in shifting the RANKL/OPG ratio in favor of OPG. (1) HCA, curcumin, galangin, genistein, resveratrol, EA, geraniin, rutin, A-type pro-anthocyanidins, CADPE, delphinidin, fisetin, peonidin, honokiol, rosmarinic acid, arbutin, oleuropein, silibinin, luteolin, myricetin, EGCG, (+)-catechin, naringenin, apigenin, kaempferol, quercetin, quercitrin, formononetin, tyrosol

Different polyphenols exert their osteoprotective effects through suppressing RANKL-induced NF-κB, thus affecting osteoclast differentiation and bone remodeling (Fig. 6).

Examples are given by polyphenols such as HCA, which exert inhibitory effects on osteoclastic cells formation induced by various osteoclastogenic factors (Lai and Yamaguchi 2006b, 2007), by functioning as natural NF-κB antagonists, since they block the binding of RANKL to its receptor RANK and thus relieving the inhibitory action of TNF-α on the pro-anabolic SMAD pathway (Yamaguchi and Weitzmann 2009b; Yamaguchi 2012) (Fig. 6). Inhibition of RANKL has also been shown by treatment of human osteosarcoma cells (Lin et al. 2014) with *Punica granatum* fruit extract and in primary BMSCs (Oh et al. 2008) and osteoclast precursors (Huh et al. 2013) with curcumin and galangin (a flavonol found in *Alpinia officinarum*), thus inhibiting osteoclast formation. In an in vivo study involving women in postmenopause, intake of genistein downregulated RANKL expression and secretion, thus decreased RANKL/OPG ratio (Marini et al. 2008). In addition, dried plum polyphenols, at the doses of 5 and 10 mg/l, also act on bone resorption, by down-regulating RANKL expression and by directly suppressing osteoclast differentiation and activity via lowering TNF-α and NO production (Bu et al. 2009). Contrasting results show involvement of resveratrol on RANKL production, in fact Boissy (Boissy et al. 2005) and Shakibaei (Shakibaei et al. 2011) suggest reduction of RANKL levels at 100 and 5 μM, respectively, while in the study from Casarin and al. (Casarin et al. 2014), the daily administration of 4.4 × 10^−5^ mol/kg resveratrol had no significant effect on the reduction of RANKL.

Although EA has been shown to reduce NF-κB in macrophage cells (Spilmont et al. 2013), different results demonstrated that the effect of EA could be NF-κB-independent (Rogerio and Favarin 2013), as it does not reduce NF-κB activation during the peak of inflammation. Geraniin and rutin have been shown to have osteoprotective effects (Lu et al. 2015) by exerting inhibitory actions on osteoclastogenesis and osteoclast functions (He et al. 2013), through mechanisms mediated via suppression of diverse signaling pathways, including NF-κB and TNF-α (Pan et al. 2000; Kyung et al. 2008), while A-type proanthocyanidins do inhibit osteoclast differentiation (Tanabe et al. 2011), LPS-induced MMPs production and biofilm formation and modulate inflammatory responses to periodontopathogens, by negatively regulating the DNA-binding activity of NF-κB p65 (La et al. 2009a).

CADPE is a specific inhibitor of NF-κB induced by different inflammatory agents, such as TNF and H_2_O_2_ (Natarajan et al. 1996), shown to have a beneficial effect on bone healing, following an inflammatory reaction induced by in vitro irradiation (Linard et al. 2004). One of the molecular mechanisms proposed to explain CADPE inhibitory activities is represented by its ability to covalently modify sulfhydryl groups of the NF-κB subunits, thus affecting NF-κB binding to DNA (Natarajan et al. 1996; Marquez 2003), without influencing IκB degradation. Furthermore, NF-κB is crucial for the early stages of RANKL-induced osteoclastogenesis and treatment of macrophages and osteoclasts with 10 μM CADPE, showed inhibition of NF-κB activation and apoptosis and downregulation of the osteoclastogenesis-related genes NFATc1 (Marquez 2003) and c-fos (Ha et al. 2009), thus making this polyphenol a useful compound for the treatment of osteolytic bone diseases (Ang et al. 2009) (Fig. 6). Also delphinidin and fisetin have potent inhibitory effects on bone resorption, with the involvement of NF-κB pathway, by downregulating c-fos and NFATc1 (Choi et al. 2012; Léotoing et al. 2013; Moriwaki et al. 2014). Conversely, peonidin, an anthocyanin from *Vaccinium macrocarpon*, has been shown to have no influence on osteoclast differentiation (Moriwaki et al. 2014), demonstrating a structure-related different mechanism of action on the skeletal system.

Honokiol, one of the major active ingredients of *Magnolia* extract, is reported to inhibit osteoclast differentiation in a dose-dependent manner (0.1–100 µM) through a mechanism involving suppression of TNFα-induced NF-κB activation, by inhibiting p65 nuclear translocation and by intensifying IκB stabilization and alleviation of the repressive action of TNFα on SMAD signaling (Yamaguchi 2011).

Rosmarinic acid, arbutin, oleuropein (isolated from *Olea europaea* olive oil) and polyphenols from *Punica granatum* fruit peel extract are able to inhibit bone resorption by blocking mRNA expression of osteoclast marker genes, such as MMP-9, cathepsin-K, calmodulin, C-C chemokine receptor type 2 (CCR2), calcitonin receptor and TRAP, via downregulating NF-κB, hence also NFATc1, thus affecting osteoclast activity and differentiation at an early stage (Hsu et al. 2011; Santiago-Mora et al. 2011; Omori et al. 2015; Spilmont et al. 2015). Silibinin, the major active constituent of the natural compound silymarin (the isomeric mixture of flavonolignans extracted from *Silybum marianum*), inhibits osteoclastogenesis by negatively targeting multiple osteoclast specific signaling molecules, in particular NFATc1 and its related downstream genes, such as TRAP, cathepsin K and osteoclast-associated immunoglobulin-like receptor (OSCAR). In parallel, it inhibited RANKL-induced DNA binding of NF-κB and AP-1 (Kim et al. 2009; Kavitha et al. 2014).

Luteolin has been characterized as a natural compound, whose properties have inhibitory effect upon osteoclast resorptive activity, some indicating, as possible target, osteoclast differentiation with inhibition of RANKL-induced signaling pathway and inhibition of the expression of NFATc1 gene (Lee et al. 2009; Kim et al. 2011a; Shin et al. 2012), while some others do not (Crasto et al. 2013). The same situation has been seen following treatment, on osteoclast precursors and mature osteoclasts, with 10–30 mg/l dried plum (DP) polyphenols which, on one hand, exert their benefic actions on bone metabolism by decreasing, at osteoclastogenesis through a mechanism involving NFATc1 and through suppression of inflammatory mediators, such as NO and TNF-α and, on the other hand, it elevates TNF-α levels in macrophages. It is, therefore, clear that the different effects, probably due to the different types and concentrations of phenolic compounds, seen in the two cell lineages are cell-type dependent (Bu et al. 2008).

Fisetin’s action against bone resorption has been seen to be primarily elicited on osteoclastogenesis, at 10 μM, by inhibiting NFATc1 and c-Src, as well as AP-1/c-fos (Sakai et al. 2013).

Curcumin has been extensively studied because of its ability, at doses ranging from 40 to 60 μM, to inhibit NF-κB activation (Bharti and Donato 2003; Guimarães et al. 2011) and AP-1 activation induced by inflammatory stimuli, such as IL-1β, TNF-α (Aggarwal 1995) and RANKL (Bharti et al. 2004), by keeping the NF-κB/IκB complex inactivated in the cytoplasm (Jobin et al. 1999; Bharti and Donato 2003; von Metzler et al. 2009), thus suppressing subsequent transcription of pro-inflammatory genes, such as TNF-α, IL-6 (Zhou et al. 2013), cyclooxygenase 2 (COX2), vascular endothelial growth factor (VEGF) (Csaki et al. 2009) and iNOS (Chowdhury et al. 2008) and contributing to inhibition of MMPs synthesis (Kumar et al. 2012). This anti-inflammatory property of curcumin has also effect on osteoclastogenesis, in which cytokine production is associated with regulation of osteoclast formation and function. In this regard, curcumin has been indeed shown to induce apoptosis in osteoclasts, which possible mechanism has been hypothesized to be correlated with inhibition of NF-κB (Hall et al. 1995; Ozaki et al. 1997) and by decreasing RANKL expression (Zhou et al. 2013), although Hie et al. (2009). showed osteoclastogenesis to be inhibited through suppressing expression of c-fos and c-jun, rather than RANK, in vivo.

A combination of genistein (1 μM) and zinc (10 μM) has been shown to stimulate osteoclast apoptosis through a mechanism involving caspase-3 activation and to suppress osteoclastogenesis through downregulating NFATc1 expression (Uchiyama and Yamaguchi 2007a), while in osteoblastic cells their combination (10 and 100 μM, respectively) resulted in enhanced mineralization through enhancement of protein synthesis, by activating aminoacyl-tRNA synthase (Uchiyama and Yamaguchi 2007b). Myricetin, at 10 μM, has been demonstrated to inhibit inflammatory cytokine-mediated apoptosis of osteoblasts, by preventing Fas upregulation and by increasing the expression of the antiapoptotic FLICE (FADD-like IL-1β-converting enzyme)-inhibitory protein (FLIP) (Kuo 2005). Myricetin action is also elicited through suppressing the MAPK signaling pathways (Ko 2012; Wu et al. 2015a), as well as NF-κB, thus inhibiting RANKL-induced osteoclastogenesis (Wu et al. 2015a).

Besides their primary role in osteogenesis, catechins are also implicated in diminishing bone resorption: EGCG, in fact, increases osteoclast apoptosis by stimulating the DNA damage response and caspase-3 and by decreasing RANKL-induced NF-κB activation (Lin et al. 2009; Lee et al. 2010a; Jin et al. 2011). Catechins, at 40–60 mg/l, have also been demonstrated to induce apoptosis in osteosarcoma cells, by suppressing IκB kinase (IKK) activation and by increasing phosphorylation of IκB-α, thus inhibiting NF-κB (Hafeez et al. 2006). The consequence is that the ratio Bax/Bcl-2 shifts towards apoptosis.

The osteoanabolic effects of (+)-catechin have also been demonstrated by the increase of survival and activity of osteoblasts. Such inhibition of apoptotic cell death in osteoblastic cells may result from the decrease in production of TNF-α and IL-6, thus increasing survival and ALP activity at a dose of 10 μM (Choi and Hwang 2003). Catechins, at 30 μM, have also been shown to be involved in the suppression of bone resorption, through acting on osteoblasts, by inhibiting the synthesis of genes associated with bone resorption, such as RANKL, COX-2, microsomal prostaglandin E synthase (mPGES)-1 and mPGES-2 (Tominari et al. 2015).

Given that NF-κB plays a pivotal role by coordinating the induction of a wide range of genes encoding pro-inflammatory cytokines [e.g., IL-1, IL-2, IL-6, and tumor necrosis factor receptor (TNFR)], chemokines (e.g., IL-8, macrophage inflammatory protein (MIP)-1R, and monocyte chemotactic protein (MCP)-1, adhesion molecules [e.g., intercellular adhesion molecule (ICAM), vascular cell adhesion molecule (VCAM), and E-selectin], acute-phase proteins (e.g., COX-2, iNOS, etc.), it is very likely that the molecular mechanism implicated by polyphenols in attenuating inflammation is represented by NF-κB inhibition.

In fact, several studies (Kohyama et al. 1997; Pan et al. 2000; Bertelli et al. 2002; Maiuri et al. 2005; Puel et al. 2006; De Stefano et al. 2007; Puel et al. 2008; Su et al. 2015) report the ability of phenolic compounds to be effective in inhibiting inflammatory cytokines involved in the acute phase of inflammation, but also in enhancing anti-inflammatory cytokines, such as IL-10 (Comalada et al. 2006), targeting macrophagic cells and osteoblasts.

Catechins, naringenin and apigenin also target osteoclastogenic cytokines, as they downregulate IL-1, IL-23, MCP-1, MCP-3, regulated on activation, normal T cell expressed and secreted (RANTES) and IL-6, as well as RANKL expression (Bandyopadhyay et al. 2006; La et al. 2009b), through inhibiting NF-κB activation (Ishida et al. 2007; Nakamura et al. 2010) and, so, contributing to impairing osteoclastogenesis (Lee et al. 2010a). On the contrary, EGCG, in the range between 0.05 and 0.1 mol/l, has been seen to enhance IL-1 stimulated IL-6 release by osteoblastic cells, by blocking the AMPK-IκB/NF-κB pathway, thus having a role in bone remodeling mediation, being IL-6, in addition to a potent bone resorptive cytokine, also an osteotropic factor that modulates bone remodeling (Kuroyanagi et al. 2013).

Naringenin molecular mechanism of action has been investigated and the results show that it diminishes NF-κB expression (Tsai et al. 1999; Kanno et al. 2006; Ang et al. 2011), it inhibits RANKL-induced p38 signaling and NFATc1 transcriptional activity, thus suppressing the expression of inflammatory genes, such as iNOS, COX-2, TNF-α and IL-6, regulators of osteoclastogenesis and osteoclast differentiation (Wang et al. 2014a). Estrogen receptor-independent actions on osteoblast, such as inhibition of TNF-α-induced secretion of IL-6 and MCP-1, have been shown to be exerted by kaempferol, at 10 μM, through avoiding nuclear translocation of NF-κB (Pang et al. 2006).

A reduced expression of inflammatory molecules, such as IL-1β, TNF-α and IL-17, following quercetin treatment (3.3 × 10^−4^ mol/kg), has been shown to also negatively affect RANKL expression and downregulation of the adhesion molecule ICAM-1 in a mouse periodontitis model (Napimoga et al. 2013). Green tea polyphenols decrease inflammatory mediators such as COX-2, TNF-α (Shen et al. 2010), IL-1α, IL-2, IL-4, IL-10, granulocyte-macrophage colony-stimulating factor (GM-CSF) and interferon γ (IFNγ) (Shen et al. 2012), while quercitrin has also been demonstrated to exert anti-resorbing effects thanks to its anti-inflammatory properties in human gingival fibroblasts, by reducing IL-6 and MMP-1 expression (Gómez-Florit et al. 2014, 2015), responsible for induction of bone resorption and extracellular matrix (ECM) degradation, respectively (Gómez-Florit et al. 2014). Formononetin is able, at the concentration range 1–10 μM, to inhibit osteoclast differentiation by downregulating RANKL-induced production of cytokines and chemokines through suppressing phosphorylation of the NF-κB subunit p65 and IκBα degradation, as well as downregulating Akt and MAPKs, thus negatively affecting c-fos and NFATc1 expression (Huh et al. 2014).

Moreover, apigenin is also able to inhibit IFNγ-stimulated chemokine (C-X-C motif) ligand (CXCL)-9 and CXCL-10 secretion, as well as secretion of leptin, thus negatively regulating osteoclastogenesis (Bandyopadhyay et al. 2006; Goto et al. 2015).

## Redox signaling pathway

Oxidative stress is a disequilibrium between the production of reactive oxygen species and antioxidant defenses, which may lead to tissue injury (Halliwell 1994). Free radicals are the byproducts of many metabolic pathways, from reactions involved in photosynthesis and respiration, but also in response to external electromagnetic stimuli. Namely, any chemical species that contains unpaired electrons is defined as free radical. Examples include ROS and reactive nitrogen species (RNS). Thanks to their potent antioxidant properties, polyphenols, besides negatively targeting inflammatory cytokines, do exert inhibition of bone resorption by also enhancing the levels of the antioxidant defense system, raised against ROS and other free radicals, therefore making them promising molecules to be employed in oxidative stress situations, such as after tooth extraction (Al-Obaidi et al. 2014a, b).

EA, together with other polyphenols, has been found to exert a preventive action on bone loss, by acting on oxidative stress biomarkers (Sellappan and Akoh 2002).

As an antioxidant, curcumin 5 μM does prevent production of ROS—which are responsible for activation of NF-κB—by regulating expression of genes implicated in RANKL-induced osteoclast differentiation. Therefore, by suppressing NF-κB signaling, curcumin indirectly and negatively acts on NFATc1 gene expression, thus resulting in inhibition of the osteoclast differentiation process (Moon et al. 2012). Moreover, thanks to its ROS scavenger activity, curcumin is able to dose dependently (0.5–4 μM) up-regulate the content of antioxidant enzymes such as glutathione peroxidase (Gpx)-1, in the osteoclast, thus modulating ROS levels (Kim et al. 2011b). Curcumin, at 10 μM, is also an inducer of HO-1 expression, which contributes to give increased resistance to oxidative stress and plays an important role for bone marrow stem cell differentiation in the osteoblastic lineage (Gu et al. 2012). Contrasting results show, however, the lack of inhibition of bone resorption in different works (Guimarães et al. 2011, 2012). Moreover, treatment of MG-63 osteoblastic cells with curcumin 20–30 μM (Moran et al. 2012) elicits inhibition of proliferation, accordingly to a study (Notoya et al. 2006) in which the presented results show that curcumin 5 μM inhibited the proliferation and metabolism of osteoblasts via suppression of the activation of AP-1.

Thanks to their ROS scavenging activity, also icaritin (a flavonoid isolated from *Epimedium pubescens*) and phloredzin exert an inhibitory effect on bone resorption by reducing superoxide generation in osteoclasts (Huang et al. 2007), through decreasing PGE_2_ production by inhibiting COX-2 and hypoxia-inducible factor 1-alpha (HIF-1α) pathways (Puel et al. 2005; Hsieh et al. 2011). Quercetin and quercitrin also inhibit osteoclastogenesis through downregulating COX-2 expression (Guo et al. 2012c) and NO synthase (Gómez-Florit et al. 2015), given that NFATc1 is a transcription factor (TF) responsible for the translation of many genes, including cytokines, cell surface receptors and enzymes such as COX2.

Genistein antioxidant properties are important in controlling ROS generation and, thus, in protecting the disruption of the mitochondrial electron transport chain system by downregulating NADPH oxidase (Nox)-1 expression in a dose-dependent manner (1–10 μM), as well as Nox-1 activation via TNF receptor associated factor (TRAF)-6/cSrc/PI3K signaling pathway in RANKL-mediated osteoclast differentiation (Lee et al. 2014a). Furthermore, this scavenging effect is also demonstrated by the upregulation of Nrf2, a nuclear factor that contributes to the enhanced production of antioxidant enzymes such as SOD-1 and HO-1 (Lee et al. 2014a). Furthermore, also fisetin, at 10 μM, suppresses RANKL-induced ROS formation by enhancing the expression of various Nrf2-mediated oxidative stress-response enzymes (Sakai et al. 2013).

Resveratrol, thanks to its antioxidant properties, is able to restore enzymes of the antioxidant defense system, such as catalase (CAT), SOD and glutathione peroxidase (GPx) in a dose-dependent manner (10, 30, 90 μM), by restoring the normal levels of forkhead box O (FoxO)-1 and by inhibiting the phosphorylation of p66shc (Zhao et al. 2015). Furthermore, DP bone anabolic effects elicited following administration of 25% (w/w) in C57BL/6 mice for 4 or 12 weeks, also include enhancement of glutathione peroxidase, suggesting the involvement of antioxidant mechanisms (Smith et al. 2014).

Moreover, myricitrin, a glycoside from myricetin, is able, at 1–10 μM, to inhibit bone-resorbing cytokines production under oxidative conditions, showing protective effects against osteoblast cytotoxicity, thanks to its antioxidant properties (Huang et al. 2014). The authors suggest a molecular mechanism to explain these protective effects, involving FoxO signaling in osteoblasts.

Being EGCG an iron ion chelator, its reductive action on Fe(III) is involved in osteoclast apoptosis catalyzed through the Fenton reaction, which leads to production of hydroxyl radicals—potent reactive oxygen species—responsible for a direct cleavage of DNA and caspase-3 activation in osteoclasts (Islam et al. 2000; Nakagawa et al. 2002; Yun et al. 2007) (Fig. 6). Thus, reduction of oxidative stress by catechins promotes osteogenic effects, by inhibiting osteoclastogenesis and bone resorption (Zeng et al. 2014).

Protective antioxidant actions on osteoblastic cells are exerted by apigenin flavone 1 μM, with positive antioxidant actions on osteoblast differentiation, survival and function, through enhancing the cell survival-related molecular pathways PI3K, Akt and ERK2 and through upregulating the expression of the antioxidant enzymes SOD-1, SOD-2 and GPx (Jung 2014). Conversely, reports show that apigenin does not exhibit antioxidant effects on osteoblastic cells and that, instead, it both inhibits osteoblastogenesis and osteoclastogenesis (Hagiwara et al. 2011; Goto et al. 2015). Proanthocyanidins, instead, exert a significant osteoblast protection by ameliorating the H_2_O_2_-induced mitochondrial dysfunction effect at 1 μM, by inhibiting their apoptosis through suppressing the activation of p53 signaling (Zhang et al. 2014).

## PI3K/Akt signaling pathway

Several tyrosine kinases, such as the insulin receptor and cytokine receptors, take part in the promotion of cell survival and proliferation, through activating the phosphoinositide pathway. Once activated, these receptors recruit the PI3K enzyme, which directly activated kinase is the PKB, also named Akt.

Among the several TFs activated by the PI3K/Akt pathway, NF-κB, FoxOs and cAMP response element binding protein (CREB) have been shown to have a role in regulating osteogenic pathways implicated in osteoblast differentiation. In particular, Akt activation also affects FoxO3, Runx2, Osx and activating transcription factor (Atf)-4, which are directly implicated in bone development and bone cell functions.

Cross-talk with other osteogenic signaling pathways, such as Wnt, BMP and NO/cGMP can also occur, thus contributing to enhance or maintain bone development (Guntur and Rosen 2011).

Therefore, it is not surprising that targeting of PI3K/Akt pathway by polyphenols leads to controlling of a series of mechanisms involved in cell survival, growth and proliferation and, concerning bone system, induction of osteoblast proliferation and differentiation, while inhibition of osteoclast proliferation and differentiation, resulting in an osteoanabolic effect.

Promotion of osteogenic differentiation of BMSCs by icariin is reached via enhancing activation of the PI3K-Akt-endothelial NOS (eNOS)-NO-soluble guanylyl cyclase (sGC)-cGMP-dependent protein kinase (PKG) signaling pathway, through phosphorylation of Akt at 10 μM (Zhai et al. 2014), showing an interplay between PI3K and NO pathways, the latter being an important regulator of bone formation and resorption (Saura et al. 2010).

Osteoblastic differentiation via activation of Akt signaling has also been displayed by treatment of preosteoblasts with hydroxyflavones 20 μM, which action is focused on the stimulation and the increase of ALP activity (Lai et al. 2014). In addition to stimulating osteoblastic differentiation, cajanin 1.0 × 10^−5^ μM also promotes osteoblast proliferation and activity, through activating the Akt signaling pathway (Bhargavan et al. 2009). Protection of osteoblasts from apoptosis, by inhibiting p53 and by increasing Akt phosphorylation is another effect exerted by the quercetin analogue 6-C-β-d-glucopyranosyl-(2S,3S)-(+)-3′,4′,5,7-tetrahydroxyflavanol (GTDF), isolated from *Ulmus wallichiana* (Khan et al. 2013).

In addition, EGCG significantly attenuates, in a dose-dependent manner (10–30 μM), the phosphorylation rate of Akt in osteoblasts induced by sphingosine 1-phosphate, thus inhibiting heat shock protein (HSP)-27 (Natsume et al. 2009), reported to be involved in the balance between differentiation and apoptosis (Leonardi et al. 2004). Another mechanism of action, exerted by naringin 0.01 μM, aimed at promoting bone cell proliferation, includes the recruitment of Akt, thus facilitating phosphorylation and stabilization of β-catenin (Wang et al. 2015).

On the other hand, inhibition of RANKL and IL-1-induced osteoclast differentiation, through the inhibition of Akt phosphorylation, has also been reported following treatment with (2S)-2′-Methoxykurarinone prenylflavonoid (Kim et al. 2014).

## AMPK signaling pathway

The AMPK is a signaling protein that has originally evolved to act as a sensor of energy status in mammals, being a heterotrimeric complex activated by increases in AMP:ATP ratio, which reflects dangerous metabolic stresses. AMPK activation is allosterically achieved following binding of AMP, while pharmacological activators, such as plant-derived products, which include resveratrol (12.5–50 μM) (Baur et al. 2006), berberine (Lee et al. 2006), genistein and EGCG (Hwang et al. 2005) have been shown to induce AMPK activation through an indirect mechanism, by increasing cellular AMP levels.

Besides limiting energy spending, it also plays a crucial role in growth inhibition and in blocking the cell cycle. In fact, in conditions where nutrients are scarce, AMPK acts as a metabolic checkpoint by inhibiting cellular growth, via suppression of the mammalian target of rapamycin complex (mTORC)-1 signaling (Mihaylova and Shaw 2012).

AMPK is ubiquitously expressed, but its function and regulation in bone tissue are poorly understood. However, finding out that energy metabolism affects bone remodeling, suggested that a cross-talk between these two systems exists (Confavreux et al. 2009).

First of all, adipocytes and osteoblasts share a common progenitor, that is the MSC and second, several interactions between adipocyte-derived hormones, such as leptin and adiponectin, and bone have been described (Pino et al. 2012). Furthermore, not only direct actions of these hormones on bone cells occur, but also indirect actions, through acting on receptors in the central nervous system. Several in vitro studies (Kanazawa et al. 2007, 2008, 2009; Quinn et al. 2010) show how modulation of AMPK affects bone cell differentiation and function, in particular activation of AMPK has been demonstrated to be inhibitory for osteoclast differentiation (Lee et al. 2010b), while AMPK activation in osteoblasts has been shown to be important for bone nodule formation and maintenance of bone mass (Shah et al. 2010).

Evidence for AMPK activation by polyphenols are primarily given in the field of ameliorating the negative effects of a high fat rate, via an indirect mechanism of AMPK activation, which involves either PPARγ or Sirt1, thus switching the adipogenic pathway towards the osteogenic one (Hwang et al. 2005; Yamashita et al. 2006; Zang et al. 2006).

Naringin, the major flavonoid glycoside in *Citrus paradisi*, induces bone development through recruiting, at a concentration of 0.01 μM, AMPK in osteoblasts, thus facilitating phosphorylation of β-catenin at Ser-552 (Wang et al. 2015), showing that a cross-talk between AMPK and Wnt/β-catenin pathways exists (Zhao et al. 2010a).

Moreover, activation of AMPK in osteoclast precursors by resveratrol and EGCG, suppresses osteoclast formation and bone resorption without stimulating RANKL-RANK signaling (Lee et al. 2010b; Zhou et al. 2014).

## Wnt/β-catenin signaling pathway

The evolutionarily-conserved Wnt extracellular signaling pathway is a complex network, containing numerous components, implicated in different developmental processes, such as embryogenesis and adult tissue homeostasis, but also in mitogenic stimulation, cell fate determination and differentiation (Soltanoff et al. 2009). Wnt ligands are cysteine-rich proteins with distinct effects on different cellular events and, in bone, they control chondrogenesis, osteoblastogenesis and osteoclastogenesis (Monroe et al. 2012).

**Fig. 7:**
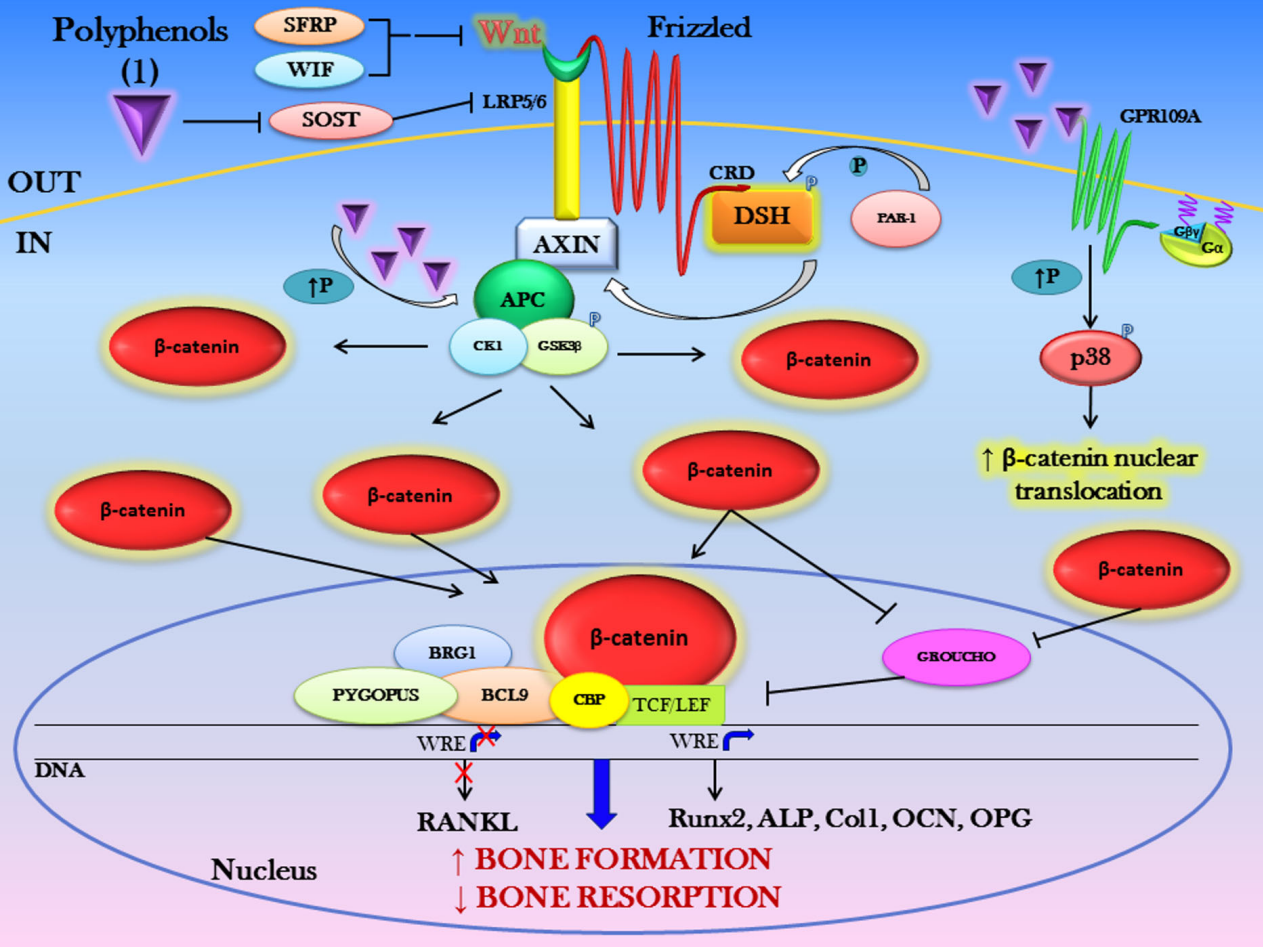
The osteogenic Wnt/β-catenin pathway and its interplay with polyphenols. Accumulation of β-catenin in the cytosol and its nuclear translocation are favored by phenolic compounds, which thus exert stimulatory effects on bone formation. Conversely, polyphenols inhibit bone resorption, through decreasing RANKL expression and relieving SOST inhibitory action on Wnt receptor. *SFRP* secreted frizzled-related protein 1, *WIF* Wnt inhibitory factor 1, *CRD* cysteine rich domain, *CK1* casein kinase 1. (1) Baicalein, myricetin, orientin, luteolin, curcumin, EGCG, resveratrol, phenolic acids

Several polyphenols have been shown to target Wnt pathway in bone, with evident stimulatory effects of osteoblast differentiation (Chen et al. 2010; Guo et al. 2012b) and inhibition of osteoclast differentiation and function (Guo et al. 2012b) (Fig. 7).

Among them, myricetin 20 μM has been demonstrated to activate the Wnt/β-catenin pathway by increasing β-catenin expression levels and TCF/LEF-driven downstream genes (Ying et al. 2014) and Luteolin 5 μM and its glycoside orientin 20 μM have been shown to reduce sclerostin (SOST) levels, inhibitor of lipoprotein receptor-related protein (LRP). Hence, this reduction of SOST does favour the Wnt canonical pathway, involved in osteoblast differentiation (Nash et al. 2014). The effects of curcumin on bone are also elicited through signaling pathways known to be involved in the growth, development and maintenance of bone tissue, such as the Wnt/β-catenin pathway. Concerning this, contrasting results show both curcumin-dependent Wnt/β-catenin activation (Chen et al. 2014a; Tiwari et al. 2014, 2015; Yang et al. 2015) and suppression (Cui et al. 2013; He et al. 2014) in different cell types, while experimental data show effective curcumin-induced restoration of Wnt/β-catenin signal in glucocorticoid-treated osteoblastic cells. Modulation of Wnt signaling has been reported to be also elicited by EGCG 25 μM, which increases ALP activity through activating β-catenin (Mount et al. 2006).

Phenolic acids, commonly found in blueberries, are able to increase osteoblastogenesis in mice fed with a dose of 5 mg/kg/day, through activating the niacin receptor GPR109A, which leads to increased phosphorylation of p38 MAPK, then to activation of Osx, Runx2 and Wnt signaling cascade (Chen and Lazarenko 2014) (Fig. 7), while treatment of Sprague–Dawley rats with resveratrol 2.0 × 10^−3^ mol/kg/day also leads to the Wnt/β-catenin pathway restoration, to the enhancement of IGF-1 mRNA levels and to suppression of the PPARγ signaling (Wang et al. 2013a), thus inhibiting adipogenesis and enhancing osteoblastogenesis. Inhibition of adipogenesis in favor of osteogenesis is also achieved by resveratrol 10 μM, through upregulation of the Wnt/β-catenin pathway and activation of Sirt1 (Zhou et al. 2009), which is a PPARγ inhibitor.

## TGF-β/BMP signaling pathway

BMPs are a group of growth factors that belong to the TGF-β superfamily. Their multiple roles vary from regulation of bone induction, maintenance and repair, to the determination of non-osteogenic embryological developmental processes and to the maintenance of adult tissue homeostasis (Chen et al. 2004).

**Fig. 8:**
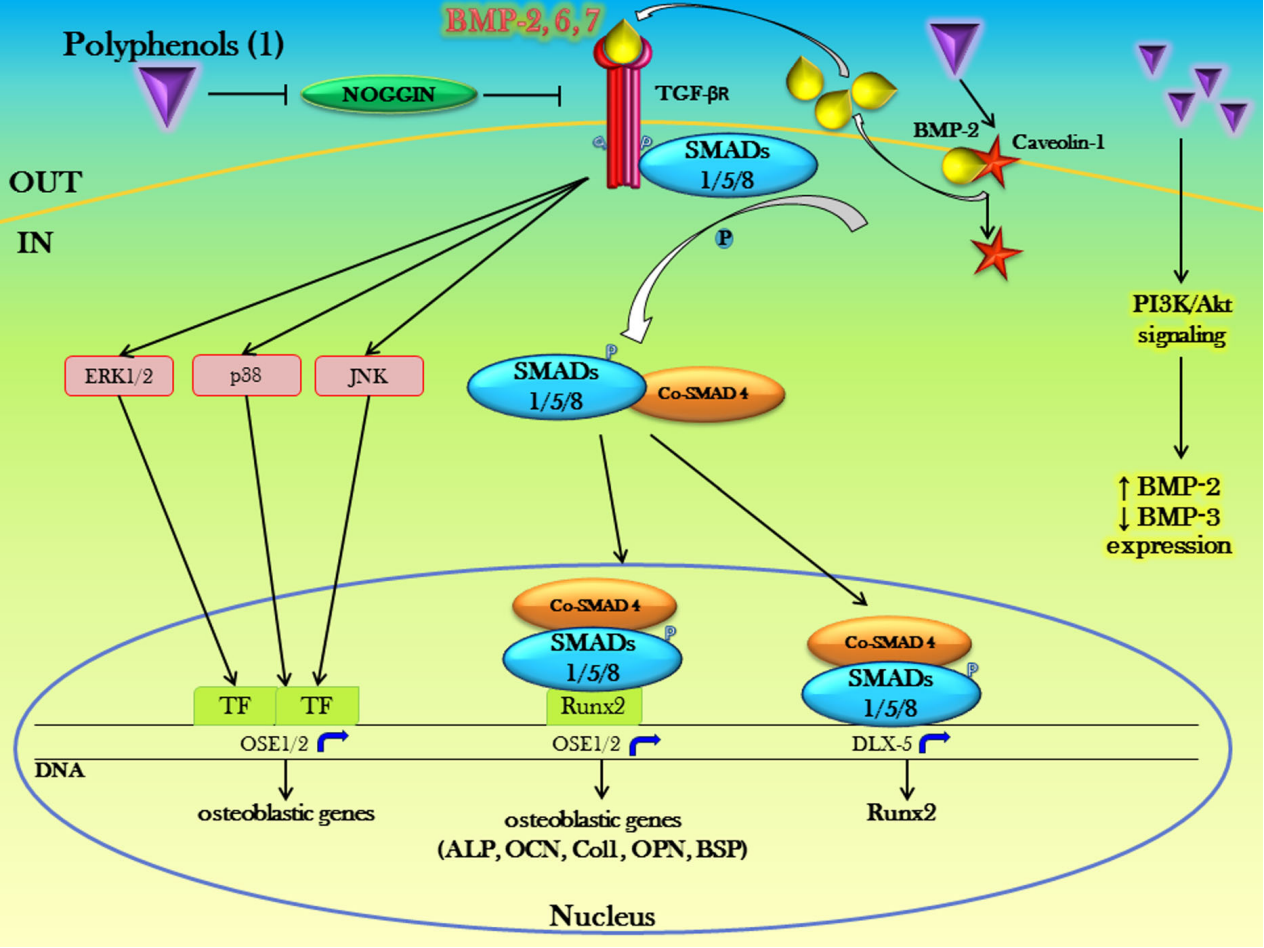
Polyphenols counteract bone disease also through BMP signaling. BMP-2, BMP-6 and BMP-7 are induced by polyphenols to activate the SMAD cascade and, so, to express osteoblastic genes important in osteoblast differentiation and function. The parallel inhibition of NOGGIN and BMP-3 expression further contributes to these osteoanabolic effects. *DLX*-*5* distal-less homeobox 5. (1) EGCG, puerarin, icariin, hesperidin, imperatorin, bergapten, syringetin, resveratrol, myricetin, apigenin, silibinin, isoquercitrin, sylimarin, piceatannol, naringin, CAFG, quercetin

Involvement of the BMP signaling in polyphenol-mediated bone anabolism has been largely investigated and several evidence show increase of new bone growth through the enhancement of the BMP-2 promoter activity and BMP-2 mRNA and protein expression (Zhang et al. 2012; Lin et al. 2014) (Fig. 8).

EGCG, in particular, has been shown to be a pro-osteogenic agent for the treatment of osteoporosis, thanks to its positive actions on osteoblast functions, on osteogenic differentiation and on mesenchymal stem cell proliferation, through the up-regulation of BMP-2 and Runx2 expression at 5 μM (Jin et al. 2014). In vitro studies show puerarin and icariin-induced BMP-2/SMAD and NO synthesis which, respectively, increase osteoblast survival, through suppressing caspase-3 activity, and positively control osteoblast differentiation and proliferation, through regulating Runx2 expression (Zhao et al. 2008; Hsieh et al. 2010; Sheu et al. 2012).

Hesperidin, the glycoside of hesperetin (a flavonone found in citrus fruit), is able to upregulate the expression of mineralization genes, such as OCN and OPN, by both stimulating BMP pathway and down-regulating Noggin signaling, thus also enhancing Runx2 and Osx expression. Moreover, c-jun and c-fos upregulation and, consequently, AP-1 positive modulation following hesperidin treatment does suggest a possible cross-talk with other signaling pathways, such as Wnt, Hedgehog and MAPKs, implicated in osteoblast differentiation (Trzeciakiewicz et al. 2010a, b). The two furocoumarins imperatorin and bergapten, the first one isolated from *Urena lobata*, the second one from *Citrus bergamia* and syringetin, from red grape, induce osteoblast differentiation and maturation through enhancing BMP-2 expression via p-38 and ERK1/2 MAPKs (Tang et al. 2008a; Hsu et al. 2009) (Fig. 8).

Given that the osteoblast lineage is a primary source for VEGF production and that VEGF plays a critical role in coupling of angiogenesis and bone remodeling, it can be postulated that resveratrol increases bone formation also through the regulation of angiogenesis. In fact, it attenuates BMP-4 and TGF-β-stimulated VEGF synthesis through suppression of the activation of p70-S6 kinase and through inhibiting p44/p42 MAPK and SAPK/JNK in osteoblasts, this inhibitory effect being mediated through SIRT1 activation (Kondo et al. 2014; Kuroyanagi et al. 2015).

Myricetin has been demonstrated to be beneficial in stimulating osteoblast activity and differentiation thanks to its ability to increase BMP-2 production and phosphorylation of SMAD1/5/8, as well as the subsequent p38 activity, thus inducing bone matrix gene expression and ALP activity in osteoblasts (Guicheux et al. 2003; Hsu et al. 2007). Apigenin at 5 and 10 μM (positively regulates osteoblast differentiation markers, through upregulating BMP-6 (Bandyopadhyay et al. 2006) and other osteoblast differentiation genes, such as ALP, OCN, OPN, bone sialoprotein (BSP), Osx and Col1 (Jung 2014). Silibinin can promote osteoblast differentiation through activating BMP signaling and, thus, SMADs phosphorylation and subsequent Runx2 signaling activation. In response to these osteogenic effects, different osteogenic markers, such as ALP, Col1 and OCN are stimulated (Kim et al. 2012b; Ying et al. 2013).

Also isoquercitrin 1 μM has been proved to be involved in osteoblastic differentiation, thanks to its ability to induce BMP-2 and, subsequently, Runx2 and ATF-6 genes to target the OCN gene (Wang et al. 2014b). Osteoblast proliferation by silymarin 10 μM has been shown by the enhancement of collagen secretion, OCN transcription and BMP pathway, which have been proved to be related to an increase of SMAD1/5/8 phosphorylation and Runx2 expression (Kim et al. 2012a).

Resveratrol 1 μM was found to induce osteogenic BMP-2 and to reduce anti-osteogenic BMP-3, thus stimulating osteoblast differentiation and maintaining the phenotype of mature osteoblasts (Su et al. 2007); resveratrol also increases OPN, BMP-2 and BMP-7, while no stimulation of BMP-6 has been observed; other resveratrol structurally-related polyphenols, like piceatannol, have instead shown the only stimulation of BMP-2 release (Gruber et al. 2003). In fact, piceatannol 1 μM increases ALP activity, OCN production and Col1 synthesis, by up-regulating BMP-2 expression both at transcriptional and translational levels, thus improving bone anabolism (Chang et al. 2006).

It has also been postulated that naringin, at 3 μM, can act through cross-talk with other signaling pathways, such as the PI3K/Akt, c-fos/c-jun and AP-1 pathways to induce osteogenic BMP-2 expression and to reduce anti-osteogenic BMP-3 (Wu et al. 2008) (Fig. 8).

Caviunin 7-*O*-[β-d-apiofuranosyl-(1-6)-β-d-glucopyranoside] (CAFG) is a non-estrogenic flavonoid glucoside extracted from leaves of *Dalbergia sissoo*, that has been proved to triggers, in ovariectomized mice fed with 1.5 × 10^−6^ mol/kg/day, BMP-2-canonical Wnt/β-catenin signaling in osteoblasts and that results in the stimulation of osteoblast differentiation and mineralization (Kushwaha et al. 2014), in fact, although BMP-2 signaling components are distinct from the known Wnt/β-catenin signal transduction pathway, they may stimulate processes that cooperate with activated β-catenin to promote osteoblast differentiation (Kushwaha et al. 2014).

Conversely, quercetin has been shown to negatively modulate TGF-β-induced or BMP-2-induced SMAD activation (Phan et al. 2004; Yamaguchi and Weitzmann 2011), thus adding further confusion about quercetin’s action on bone formation.

Moreover, although quercetin has been shown to suppress TNF-α (Zhang et al. 1996) basal and TNF-α-induced NF-κB activation (Yamaguchi and Weitzmann 2011), it exerts the same inhibitory effect both on osteoclastogenesis and osteoblastogenesis, failing to alleviate the suppressive action of TNF-α on BMP-2-induced or TGF-β-induced SMAD activation (Yamaguchi and Weitzmann 2011).

## Calcium signaling pathway

Calcium ion (Ca^2+^) is particularly important in maintaining cell homeostasis, as it participates in many cellular activities.

Thanks to its peculiar distribution, it differs from other ion functions in that its very low levels in cell cytosol enable it to act as a second messenger playing a crucial role in regulating cytosolic Ca^2+^-dependent enzymes (Ghibelli et al. 2010). Because of the presence of a large number of Ca^2+^ binding sites in the cytosol, Ca^2+^ ions are continuously buffered and, then, their movements are constantly controlled; consequently, calcium elevations can arise in specific cell zones and spread to others in a slow manner.

Calcium signal in bone is important in the regulation of osteoclast differentiation, bone resorption and gene transcription. In fact, binding of RANKL to RANK leads to production of inositol trisphosphate (IP_3_), that binds to and activates the IP3 receptor (IP_3_R), resulting in calcium release from the ER. In osteoclasts, not only signals from internal stores exist, but also signals derived from calcium entering across the plasma membrane and these cytosolic calcium oscillations are essential for the osteoclastogenesis RANKL-dependent.

The calcium released leads to activation of downstream effector proteins, such as calmodulin kinases and calcineurin. Calcineurin phosphatase dephosphorylates and, thus, activates, NFATc1 that translocates to the nucleus where it initiates the transcription of several osteoclast specific genes, such as cathepsin K, TRAP and calcitonin receptor (Hwang and Putney 2011). Concerning osteoblasts, studies report a cell sensitivity to high extracellular calcium concentrations, in that they may affect the proliferation and differentiation of osteoblasts (Farley et al. 1994; Honda et al. 1995; Eklou-Kalonji et al. 1998).

Polyphenols like resveratrol have been shown to regulate different cellular processes, by acting as ligand for transmembrane proteins, like voltage-gated calcium channels and plasma membrane calcium ATPase (Sareen et al. 2007; Sulaiman et al. 2010), but also through regulating intracellular calcium channels (Dobrydneva et al. 1999; Buluc and Demirel-Yilmaz 2006; Dobrydneva et al. 2010; McCalley et al. 2014).

The protective actions of polyphenols on bone metabolism via modulating the calcium signaling are mainly achieved through repression of bone resorption, with osteoclastogenic genes as primary targets (Yamaguchi and Sugimoto 2000; Wu et al. 2012).

A suppressive effect on bone resorption by genistein (1–10 μM) is mainly exerted through acting on osteoclast proliferation, via induction of apoptosis, through a mechanism Ca^2+^-mediated (Gao and Yamaguchi 1999b), while a repressive action on osteoclast functions by genistein 50 μM has been shown to be achieved through inhibiting inward rectifier K^+^ channels—which, in osteoclasts, are important to maintain the H^+^ transport to bone surface (Sims and Dixon 1989)—thus inducing membrane depolarization and causing [Ca^2+^]_i_ elevation (Okamoto et al. 2001).

## Discussion

Behind all the above discussed shared molecular mechanisms implied in polyphenol bone protection, it is of extreme importance to take into account how these polyvalent phytochemicals interact, influence and/or interfere with different parallel signaling pathways. One of the main properties characterizing polyphenols and, as such, the most extensively studied, is represented by their ability to exert anti-inflammatory actions by negatively regulating the inflammation pathway and, especially, its crucial NF-κB TF.

Because NF-κB gives rise to signals implicated in varied transcriptional programs with broad physiological and medical effects such as immunological response, development (Hayden and Ghosh 2004; Oeckinghaus and Ghosh 2009), survival, apoptosis and cell growth (Guttridge et al. 1999), polyphenol effects on this signaling pathway also reflect downstream of NF-κB, thanks to different crosstalks. In fact, cooperative interactions with other TFs or receptor molecules are responsible for integration of NF-κB functions with other cell-signaling pathways, thanks to different specific binding sites in the molecule itself, or on the promoters of the target genes (Perkins 2007). The estrogen receptor, in fact, may interact via protein–protein interactions with NF-κB, resulting in modulation of the binding of NF-κB to NF-κB response elements, thus regulating NF-κB-dependent gene transcription in a cell-type-specific manner and has important implications in the inflammatory processes.

For example, estrogen downregulates IL-6 production indirectly, by binding to NF-κB and thus reducing the IL-6 promoter activity (Stein and Yang 1995).

Polyphenol ability to reduce and/or suppress inflammation, also reflects on a new field of interest that is the osteo-immune-oncology, because there is a link between immunology, bone metabolism and tumorigenesis.

In this field, NF-κB is also investigated in oncogenesis, because of its ability to regulate genes involved in proliferation and apoptosis processes, with elevation of its levels in some types of cancers (Sovak et al. 1997; Reuther et al. 1998). In view of this, estrogen has been shown to inhibit different tumorigenic cell line growth, by binding to NF-κB (Pratt et al. 2003).

Other evidence of interaction between estrogen and other pathways is available in literature; for example, reciprocal effects between Wnt/β-catenin and estrogen are showed by their synergistic regulation of osteogenic differentiation (Gao et al. 2013), while, between estrogen and TGF-β, different levels of crosstalking are present, including estrogen induction of TGF-β gene expression, which results in the activation of the Smad signaling pathway and, so, in a synergistic regulation of bone metabolism (Hawse and Subramaniam 2008).

Inflammation is also linked to the development of different chronic diseases, from heart diseases, to obesity, diabetes and osteoporosis, and, furthermore, given that osteoblasts and adipocytes derive from the same MSC compartment, polyphenol action on switching differentiation towards osteoblastogenesis, rather than adipogenesis, makes these molecules a promising tool to treat metabolic dysfunctions (Ginaldi and De Martinis 2016).

Negative regulation of NF-κB by Wnt signal is also achieved through the physical interaction between β-catenin and NF-κB, that results in a minor DNA binding activity and, thus, in diminished NF-κB-related gene expression. Overexpression of β-catenin in osteoblasts, for example, leads to inhibition of NF-κB, showing a molecular connection between Wnt/β-catenin-mediated bone formation and NF-κB-mediated inflammation (Die et al. 2012). Given the ability of Wnt signaling to stimulate or suppress NF-κB pathway, opposite properties of anti-inflammation and pro-inflammation are of evidence, even if most of the results show a prevalence of the anti-inflammatory actions (Ma and Hottiger 2016).

Depending on the cellular context, these types of crosstalk can have effect of antagonism or synergism; for example, regarding BMP/Wnt relationship, the osteoblast precursor is maintained in a proliferation status by Wnt, while BMP does stimulate it to undergo the maturation stage, showing antagonism between these two pathways at a distinct developmental stage. In the subsequent developmental stages, a synergism can be observed, when the signals begin to function cooperatively (Itasaki and Hoppler 2010). However, an increment of the Wnt signal could lead to cancer initiation and progression, being β-catenin involved in carcinogenesis, especially in colorectal cancer. If it is true that many beneficial actions of polyphenols on bone metabolism are also achieved by enhancement of the Wnt signaling, it is also true that some polyphenol effects on cancer prevention are achieved by negatively targeting Wnt signaling (Amado et al. 2011). These opposite effects could be explained by the different types of polyphenols involved and, most importantly, by the doses. In fact, as already extensively reported in the previous paragraphs, different toxicity effects can be observed, for a specific polyphenol, at high (mmol/L) or low-doses (μ or nmol/L) (Williamson and Manach 2005), thus showing a dualistic phytochemical nature (Martin 2009).

By taking these observations into account, it is clear that the present understanding of how phytochemicals act on a specific biological system is still far from an effective and reproducible application in vivo.

This consideration is also supported by the observation that some polyphenols differ from others in terms of bioavailability and bioactivity.

Despite their structure diversity, they share common different properties, which are responsible for the even more growing interest in researchers and society. These multiple features, which include anti-inflammation, anti-oxidation and anti-aging, contribute to the prevention of human diseases and, specifically for the above analyzed bone system context, to the protection against bone diseases, in so far as they also include anti-resorption and pro-osteogenesis.

The many different possible chemical structures are also made more complex by the binding of sugars, such as glucose, galactose, rhamnose, xylose, rutinose, arabinopyranose and arabinofuranose to form more stable glycosylated derivatives to be stored in vacuoles and chloroplasts and by esterification with lipids and organic acids. Hence, these aspects represent an important key point that determines the degree of polyphenol bioavailability and absorption: in fact, in the small intestine, enzymes such as glycosylases are able to metabolize the glycosylated forms to aglycones, while only specific strains of the gut microbiota in the colon, are able to break the esterification bond and to generate active metabolites (Marín et al. 2015).

Besides polyphenol metabolism, other aspects to be considered are the subsequent modifications carried out by enterocytes, such as methylation, sulfation and glucuronidation, representing a detoxification mechanism by which the organism prepares the molecule to be excreted. Before polyphenols are taken to tissues or excreted through bile, urine or faeces, conjugation, mainly in the liver, with other carrier molecules or interaction with other types of polyphenols is also possible, thus increasing the factors to take into account when considering polyphenol bioavailability.

So, it is also important to investigate whether the extensive modifications which take place in the host organism, do also affect polyphenol activity, that can be different from the one that has been observed in the original sources and to notice that aglycones, the presumed active forms of polyphenols, are generally absent in plasma, although with some exceptions (D’Archivio et al. 2007).

That’s why, when considering in vivo delivery, techniques aimed at improving absorption and bioavailability should be well chosen and designed. For example, by targeting the gut bacteria genome with mutagenesis techniques to generate new and improved strains, could allow them to be more efficient in metabolizing polyphenols or to produce new or more active metabolites, while increasing the lipophilicity by combination of polyphenols with other molecules and the addiction of lipophilic groups reduces the hydrogen bonding potential. In this field, phytosomes with unique vehicle properties that arise from the complexation of a phospholipid with a phytochemical, exhibit better pharmacokinetic and pharmacodynamic profiles than free phytochemical compounds (Jain et al. 2010). Another way to improve bioavailability, involves structural changes aimed at increasing the plasma half-life (clearance) of compounds, the involvement of a vector molecule, which is directly conjugated with the polyphenol and which has affinity for a relative receptor on the target tissue, or the development of micro or nano-carriers such as microspheres, nanoparticles, microemultions and so on (Khushnud and Mousa 2013).

Regarding the engineering of delivery systems and devices, nanotechnologies, which are getting researchers more and more interested in, could represent a promising approach, as they involve the tailoring of materials at atomic level, in view of obtaining unique properties suitable for a wide variety of applications (Gleiter 2000).

Nanomedicine is a new field of treatments based on nanosystems able to enhance drugs delivery, drugs specific targeting and drugs half-life, thanks to their properties of passive accumulation in specific tissues, stability, specificity and biocompatibility. So, given the relative high clearance of polyphenols and their low stability, a nanotechnological approach aimed at increasing the phytochemical circulation in the bloodstream could represent a way to be investigated (Tabrez and Priyadarshini 2013). Furthermore, nanotechnologies specificity could be improved by creating targeted nanoparticle systems, particularly useful when the phytochemical delivery is to be obtained via topical or parenteral delivery, rather than by the classical enteral delivery. For example, considering the transdermal administration of a drug, its penetration often finds difficulties due to skin anatomy, with different routes of penetration; so, the employ of nanoparticles provide a number of advantages, including increased xenobiotic solubility and, thus, permeation, reduced toxicity, as the first-pass hepatic metabolism is avoided and high affinity for cellular membranes, due to nanoparticles surface properties (Uchechi et al. 2014).

## Conclusion

In the past few decades, accumulating data have shown potential beneficial effects of polyphenols on human health.

In particular, thanks to their multiple beneficial properties, which can modulate different signaling pathways, polyphenols are able to target the diverse bone cellular compartments, thus exerting a noticeable bone protection (Đudarić et al. 2015).

However, despite these findings, care must be taken when considering dietary intake of polyphenols, because they can act in a double manner, being dependent on many other associated factors, such as bioavailability, diet or dosage (Martin 2009) and, therefore, the need to develop tests aimed at establishing the right dose to ensure safety and the low risk of adverse effects is even more crucial.

Furthermore, besides dosage, also the form of the phenolic compound is of note, in fact it influences its bioavailability, together with the presence of intestinal microflora and gut enzymes.

So, biomedical applications of these natural compounds are severely hindered by their low bioavailability, rapid metabolism, and often by unfavorable physico-chemical properties, e.g. a generally low water solubility, as well as still insufficient scientific data derived from preclinical and clinical studies.

Given the Directive 2001/83/EC of the European Parliament and of the Council of 6 November 2001 on the Community code, relating to medicinal products for human use, at article 1, first subparagraph, the term “medicinal product” is established as “any substance or combination of substances presented as having properties for treating or preventing disease in human beings; or any substance or combination of substances which may be used in or administered to human beings either with a view to restoring, correcting or modifying physiological functions by exerting a pharmacological, immunological or metabolic action, or to making a medical diagnosis” (EC 2001).

The main purpose of the standards concerning production, distribution and intended use of medicinal products, is to ensure the protection of public health and to enable the competent authorities to make decisions on the basis of uniform tests and by reference to uniform criteria, by contributing to prevent differences of view.

Although polyphenols have been shown to possess effective biological properties in the prevention, treatment and mitigation of different bone diseases, they cannot be defined as “active pharmaceutical ingredient” (API) and they are not regulated by technical dossiers involved in the definition of “medicinal products”.

Not less important, they have not yet been shown to represent a definitive cure for bone diseases.

Aiming at reducing the differences by setting them against the evolution of science, it is concluded that properties and intended use of medicinal products are diversified not only on the basis of already existing scientific data, but also by the appropriate understanding of the existing legislation.

Therefore, thanks to their beneficial osteoanabolic effects, polyphenols could be used as adjuvants in the prevention, treatment and mitigation of the osteoporotic disease, with a strict control of the dosages at which their health benefits and lack of adverse effects have been shown.

Consequently, more in vivo tests should be necessary to determine, at first, which types of intervention on molecules do improve their bioavailability, then which doses are better useful to get the desired effects, by also taking into account the toxicity aspect. Furthermore, in a medical devices context, it is important to evaluate how polyphenols combined with medical devices do act and eventually modify their properties in the different pathways, following a sterilization process, for example, or the all necessary steps aimed at ensuring an excellent result of quality control also in the post marketing activity.

## Abbreviations

AD: Adipose tissue-derived
ALPL: Alkaline phosphatase liver/bone/kidney
AMPK: Adenosine monophosphate protein kinase
AP1: Activator protein-1
API: Active pharmaceutical ingredient
ARE/EpRE: Antioxidant response element/electrophile responsive element
Atf: Activating transcription factor
ATP: Adenosine triphosphate
Bax: Bcl-2-associated X
Bcl-2: B cell lymphoma 2
BMP-2: Bone morphogenetic protein-2
BMPs: Bone morphogenetic proteins
BSP: Bone sialoprotein
Ca: Calcium
CADPE: Caffeic acid 3,4-dihydroxy-phenethyl ester
CAFG: Caviunin 7-*O*-[β-d-apiofuranosyl-(1-6)-β-d-glucopyranoside]
cAMP: Cyclic adenosine monophosphate
CCR2: C-C chemokine receptor type 2
cGMP: Cyclic guanosine monophosphate
Col1: Collagen type 1
GPR30: G protein-coupled receptor 30
Gpx: Glutathione peroxidase
GTDF: 6-C-β-d-glucopyranosyl-(2S,3S)-(+)-3′,4′,5,7-tetrahydroxyflavanol
HCA: P-hydroxycinnamic acid
HIF-1α: Hypoxia-inducible factor 1-alpha
HO: Heme oxygenase
HSP: Heat shock protein
ICAM: Intercellular adhesion molecule
IFNγ: Interferon γ
IGF: Insulin-like growth factor
IKK: Iκb kinase
IL: Interleukin
iNOS: Oxide synthase
IP_3_: Inositol trisphosphate
IP_3_R: IP3 receptor
JNK: C-Jun N-terminal kinase
LPS: Lipopolysaccharide
LRP: Lipoprotein receptor-related protein
MAPKs: Mitogen-activated protein kinases
MCP: Monocyte chemotactic protein
MIP: Macrophage inflammatory protein
MMP: Matrix metalloproteinase
mPGES: Microsomal prostaglandin E synthase
MSCs: Mesenchymal stem cells
mTORC: Mammalian target of rapamycin complex
NAD: Nicotinamide adenine dinucleotide
NCoR: Nuclear receptor co-repressor
NFATc1: Nuclear factor of activated T-cells 1
NF-κB: Nuclear factor kappa-light-chain-enhancer of activated B cells
NO: Nitric oxide
Nrf2: Nuclear factor E2-related factor 2
OCN: Osteocalcin
OPG: Osteoprotegerin
OPN: Osteopontin
OSCAR: Osteoclast-associated immunoglobulin-like receptor
Osx: Osterix
COX2: Cyclooxygenase 2
CREB: Camp response element binding protein
CREs: Camp response elements
CXCL: Chemokine (C-X-C motif) ligand
DP: Dried plum
E2: 17β-estradiol
EA: Ellagic acid
ECM: Extracellular matrix
EGCG: Epigallocatechin gallate
eNOS: Endothelial NOS
ER: Estrogen receptor
ERE: Estrogen response elements
ERK: Extracellular signal-regulated kinase
FGF-2: Basic fibroblast growth factor 2
FLICE: FADD-like IL-1β-converting enzyme
FLIP: FLICE-inhibitory protein
FoxO: Forkhead box O
GM-CSF: Granulocyte–macrophage colony-stimulating factor
GPCR: 7-Transmembrane G protein-coupled receptor
GPER: G protein-coupled estrogen receptor 1
PGE_1_: Prostaglandin E1
PGE_2_: Prostaglandin E2
PGF2α: Prostaglandin F 2α
PI3K: Phosphatidylinositol-4,5-bisphosphate 3-kinase
PKA: Protein kinase A
PKB/Akt: Protein kinase B
PKC: Protein kinase C
PGD_2_: Prostaglandin D2
PLC: Phospholipase C
PP2A: Protein phosphatase 2A
PPARγ: Peroxisome proliferator-activated receptor gamma
RANK: Receptor activator of nuclear factor kappa-B
RANKL: Receptor activator of nuclear factor kappa-B ligand
RANTES: Regulated on activation, normal T cell expressed and secreted
RNS: Reactive nitrogen species
ROS: Reactive oxygen species
Runx2: Runt-related transcription factor 2
SAPK: Stress-activated protein kinases
SERMs: Selective estrogen receptor modulators
sGC: Soluble guanylyl cyclase
Sir2: Silent information regulator 2
Sirt1: Sirtuin 1
SMAD: Small mother against decapentaplegic
SOD-1: Superoxide dismutase 1
SOST: Sclerostin
SP1: Specificity protein-1
TF: Transcription factor
TGF-β1: Transforming growth factor-β1
TNFR: Tumor necrosis factor receptor
TNF-α: Tumor necrosis factor-α
TRAF: TNF receptor associated factor
TRAP: Tartrate-resistant acid phosphatase
TRKs: Receptor tyrosine kinases
VA: Vanillic acid
VCAM: Vascular cell adhesion molecule
VEGF: Vascular endothelial growth factor
